# Technical development and validation of a clinically applicable microenvironment classifier as a biomarker of tumour hypoxia for soft tissue sarcoma

**DOI:** 10.1038/s41416-023-02265-3

**Published:** 2023-04-21

**Authors:** Laura J. Forker, Becky Bibby, Lingjian Yang, Brian Lane, Joely Irlam, Hitesh Mistry, Mairah Khan, Helen Valentine, James Wylie, Patrick Shenjere, Michael Leahy, Piers Gaunt, Lucinda Billingham, Beatrice M. Seddon, Rob Grimer, Martin Robinson, Ananya Choudhury, Catharine West

**Affiliations:** 1 Translational Radiobiology Group, Division of Cancer Sciences, The Oglesby Cancer Research Building, The University of Manchester, Manchester Academic Health Science Centre, 555 Wilmslow Road, Manchester, M20 4GJ UK; 2 Department of Clinical Oncology, The Christie NHS Foundation Trust, Wilmslow Road, Manchester, M20 4BX UK; 3 Department of Histopathology, The Christie NHS Foundation Trust, Wilmslow Road, Manchester, M20 4BX UK; 4 Department of Medical Oncology, The Christie NHS Foundation Trust, Wilmslow Road, Manchester, M20 4BX UK; 5 Cancer Research UK Clinical Trials Unit, Institute of Cancer and Genomic Sciences, University of Birmingham, Edgbaston, Birmingham, B15 2TT UK; 6 Department of Oncology, University College London Hospitals NHS Foundation Trust, 1st Floor Central, 250 Euston Road, London, NW1 2PG UK; 7 Department of Orthopaedic Oncology, Royal Orthopaedic Hospital NHS Foundation Trust, Bristol Road South, Northfield, Birmingham, B31 2AP UK; 8 Department of Oncology, Academic Unit of Clinical Oncology (Cancer Clinical Trials Centre), Weston Park Hospital, Whitham Road, Sheffield, S10 2SJ UK

**Keywords:** Cancer microenvironment, Prognostic markers, Sarcoma, Tumour biomarkers

## Abstract

**Background:**

Soft tissue sarcomas (STS) are rare, heterogeneous tumours and biomarkers are needed to inform management. We previously derived a prognostic tumour microenvironment classifier (24-gene hypoxia signature). Here, we developed/validated an assay for clinical application.

**Methods:**

Technical performance of targeted assays (Taqman low-density array, nanoString) was compared in 28 prospectively collected formalin-fixed, paraffin-embedded (FFPE) biopsies. The nanoString assay was biologically validated by comparing to HIF-1α/CAIX immunohistochemistry (IHC) in clinical samples. The Manchester (*n* = 165) and VORTEX Phase III trial (*n* = 203) cohorts were used for clinical validation. The primary outcome was overall survival (OS).

**Results:**

Both assays demonstrated excellent reproducibility. The nanoString assay detected upregulation of the 24-gene signature under hypoxia in vitro, and 16/24 hypoxia genes were upregulated in tumours with high CAIX expression in vivo. Patients with hypoxia-high tumours had worse OS in the Manchester (HR 3.05, 95% CI 1.54–5.19, *P* = 0.0005) and VORTEX (HR 2.13, 95% CI 1.19–3.77, *P* = 0.009) cohorts. In the combined cohort, it was independently prognostic for OS (HR 2.24, 95% CI 1.42–3.53, *P* = 0.00096) and associated with worse local recurrence-free survival (HR 2.17, 95% CI 1.01–4.68, *P* = 0.04).

**Conclusions:**

This study comprehensively validates a microenvironment classifier befitting FFPE STS biopsies. Future uses include: (1) selecting high-risk patients for perioperative chemotherapy; and (2) biomarker-driven trials of hypoxia-targeted therapies.

## Background

Soft tissue sarcomas (STS) are a rare group of tumours compromising >50 malignant, heterogeneous subtypes [1]. Surgery is the cornerstone of potentially curative treatment in localised disease and the combination of wide excision and radiotherapy has excellent local control rates (80–90%) [2]. However, 50% of high-grade patients develop metastatic disease [3], which carries a poor prognosis with a median survival of ~18 months [4].

The role of neoadjuvant/adjuvant anthracycline-based chemotherapy in preventing metastatic relapse is controversial, as many large trials failed to demonstrate a consistent overall survival (OS) benefit [5, 6]. Recently, it was reported that chemotherapy may be advantageous in high-risk patients based on clinical factors (Sarculator nomogram predicted overall survival [pOS] <60% [7]). Despite differences in response to treatment between the histologic subtypes, trials are often ‘all-comer’ designs. As each subtype is extremely rare, biomarkers of adverse microenvironmental features present across subtypes might be more successful in selecting high-risk patients for clinical trials.

For high risk, localised STS surgery and radiotherapy is recommended as standard of care [8]. Optimal timing of radiotherapy is uncertain; in limb STS neoadjuvant radiotherapy gives equivalent local control to adjuvant with less long-term toxicity, but at the expense of greater wound healing complications [9]. A wide range of responses to neoadjuvant radiation has been reported in surgical specimens [10, 11], suggesting that for some patients this would delay definitive surgery with no benefit. Recently, it was reported that neoadjuvant radiotherapy was of no benefit for retroperitoneal STS [12].

The major unmet clinical needs in STS are to: (1) determine which patients are at high risk of metastatic relapse and would be more likely to benefit from systemic therapy in the neoadjuvant/adjuvant setting; (2) develop biomarkers to aid clinical decision-making with regards to neoadjuvant or adjuvant radiotherapy; (3) expand the range of systemic therapies available; and (4) improve the efficacy of radiotherapy with new radiotherapy–drug combinations.

Tumour hypoxia is an adverse microenvironmental feature of solid tumours, which promotes metastasis [13], resistance to chemotherapy [14] and radiotherapy [15], genome instability [16] and immune evasion [17]. It has been associated with adverse outcomes in STS in cohorts involving multiple subtypes [18, 19]. It is potentially targetable via a range of strategies, including hypoxic radiosensitisation, hypoxia-targeted pro-drugs and molecular targeting of downstream processes [20]. ‘All-comer’ designs for clinical trials of hypoxia-targeted therapy have been unsuccessful [21, 22]. In head and neck cancer, hypoxia-associated gene signatures can predict benefit from the addition of hypoxia-targeted therapy to radiotherapy [23, 24]. We previously derived and validated a 24-gene hypoxia-associated signature for STS that was prognostic in multiple cohorts containing a range of histologic subtypes [25].

This study aimed to (1) develop a targeted assay to measure the signature in routine pre-treatment biopsies for use in clinical trials in STS; and (2) validate the technical, biological and clinical performance of the assay in two large radiotherapy-treated cohorts, including the Phase III VORTEX trial [26].

## Methods

### In vitro hypoxia experiments

The soft tissue sarcoma (STS) cell lines HT1080 and SKUT1 were purchased from the American Type Culture Collection (ATCC, Teddington, Middlesex, UK), and cultured in Eagle’s minimum essential media (Gibco, ThermoFisher Scientific, Loughborough, UK) plus 10% foetal bovine serum (Sigma Aldrich, Gillingham, UK) under 5% CO_2_ in keeping with the manufacturer’s recommendations. Cell lines were authenticated by the Promega Powerplex 21 System (Promega UK Ltd., Southampton, UK) and underwent mycoplasma screening (Molecular Biology Core Facility, CRUK Manchester Institute, UK).

Cells were seeded in 75-cm^2^ flasks at an appropriate density to achieve 60% confluence after 48 h culture under 21% oxygen for each individual cell line. Cells were cultured under 21% oxygen for 24 h, after which the media was changed prior to a further 24 h culture under 21%, 1% or 0.2% oxygen (Whitley H35 Hypoxystation, Don Whitley Scientific, Bingley, UK). Experiments were repeated for three different passages for each cell line. Hypoxia-exposed cells were harvested under hypoxia.

### Clinical cohorts

Clinical assay development and biomarker validation of the 24-gene hypoxia signature was performed in four cohorts of adults with STS: (1) Manchester Cancer Research Centre (MCRC) biobank (*n* = 34) (18/NW/0092); (2) a single centre Manchester retrospective cohort (*n* = 165) (06Q1403256); (3) the VORTEX-Biobank cohort (*n* = 203) (06/MRE03/3, NCT00423618); and (4) an intra-tumour heterogeneity cohort (*n* = 10 tumours, *n* = 45 biopsies, 3–8 biopsies/tumour) (06Q1403256). Baseline clinical characteristics for all cohorts and RNA quality control (QC) metrics are summarised in Supplementary Tables 1–4. Further details regarding the clinical cohorts are included in the Supplementary Methods online.

VORTEX was a Phase III, randomised, controlled trial comparing radiotherapy volumes in 216 randomised patients. The VORTEX-Biobank (tissue collection for transcriptomic, genomic and proteomic profiling) and hypoxia signature study were pre-planned translational elements of the main VORTEX trial. The VORTEX and Manchester validation cohorts consisted of patients with extremity STS, which was mostly high-grade (~85%).

The Sarculator prognostic nomogram app (https://apps.apple.com/us/app/sarculator/id1052119173) was used to calculate 10-year pOS. A cut-off of ≤60% 10-year pOS was used to define Sarculator high risk as per the re-analysis of EORTC-STBSG 62931. Protein expression of HIF-1α and CAIX was determined in formalin-fixed paraffin-embedded (FFPE) tumour samples by immunohistochemistry (IHC) and scored by a sarcoma pathologist (PS) as described previously [19].

### RNA extraction

RNA was extracted from cell lines using the RNeasy Midi Kit (75144, Qiagen, Manchester, UK). RNA was extracted from 10-µm sections from FFPE tumour samples using either the High Pure FFPET RNA Isolation Kit (06650775001, Roche, Welwyn Garden City, UK) (Manchester, VORTEX-Biobank and heterogeneity cohorts) or the FFPE RNA/DNA Purification Plus Kit (54300, Norgen Biotech Corp.) (MCRC biobank cohort).

Nucleic acids were measured by a NanoDrop One and Invitrogen Qubit 4 Fluorometer (ThermoFisher Scientific, UK) for quantity and quality (absorbance ratios) parameters. RNA integrity number (RIN) and DV200 (percentage of fragments >200 bp) were determined using an Agilent bioanalyzer (Agilent Technologies, Stockport, UK).

### Targeted assay endogenous control gene selection

Seven candidate endogenous control genes were chosen for inclusion in the TLDA and nanoString targeted assay designs. These were the most stably expressed genes (lowest coefficient of variation) in two STS cohorts with whole transcriptome gene expression data (The Cancer Genome Atlas [TCGA] cohort, *n* = 258 and the VORTEX-Biobank *n* = 70). The candidate genes were assessed in the MCRC biobank cohort using GeNorm [27], a publicly available excel macro designed to identify suitable control genes. A low M-value represents low variability in the ratio of gene expression between endogenous control genes across the test samples. The lowest pairwise variation in M-value between sequentially calculated normalisation factors when further genes were added was seen with the use of six endogenous control genes (pairwise variation = 0.11) for TLDA and five for nanoString (pairwise variation = 0.10). A pairwise variation of <0.15 is recommended.

### TaqMan array cards (TLDA)

Custom 384 well microfluidic TaqMan low-density array (TLDA) cards (Life Technologies, Paisley, UK) with each well containing a single TaqMan assay were designed for the 24-gene signature and seven candidate endogenous control genes. RNA was reverse transcribed, pre-amplified and then run on TLDA cards on the QuantStudio 12 K Flex Real-Time PCR System (Life Technologies) according to the manufacturer’s protocol. Cycle threshold (Ct) values were exported from the Thermofisher cloud (ThermoFisher Scientific, Loughborough, UK) and analysed manually. Further details in Supplementary Methods online.

### NanoString

NanoString codesets were designed (NanoString Technologies, Seattle, WA, USA) to include the 24-gene signature and seven candidate endogenous control genes (five endogenous control genes for the final assay). Samples were hybridised and then processed on the nCounter Prep Station (NanoString Technologies) and imaged on the nCounter Digital Analyzer (NanoString Technologies) according to the manufacturer’s protocol. Data quality control and normalisation was performed using nSolver analysis software 4.0 (NanoString Technologies). Further details in Supplementary Methods online.

### Hypoxia class prediction

Yang et al. [25] had previously defined a 24-gene hypoxia signature for STS. The original training data from this study was used to generate a PAMR model (R package pamr v 1.56.1) [28] with hypoxia-low and hypoxia-high centroids for the 24-gene signature. For clinical deployment, no shrinkage was applied to the centroids and hypoxia class predictions were based on the shortest Spearman distance to the unshrunken centroids for median-centred data from each sample. The signature result is binary; hypoxia low or hypoxia high.

### Statistical analyses

All analyses were performed in either GraphPad Prism Version 8.0.2 or R programming language (v 3.6.1, Vienna, Austria). The survival package (v 3.1–12) was used to perform Cox regression analysis (Cox proportional hazards model) to provide hazard ratios (HR) and 95% confidence intervals (CI) in univariable and multivariable analyses. For studies of the 24-gene signature, clinical outcome measures included local recurrence-free survival (LRFS), metastasis-free survival (MFS), disease-free survival (DFS) and overall survival (OS) times. For the retrospective cohort this was defined as time from the first sarcoma clinic referral to event, and for the VORTEX-Biobank cohort this was defined as the time from randomisation to the event. Patients without an event were either censored at the date of the last follow-up or at 5 years, whichever was earlier. Clinical baseline features (age, sex, WHO PS, size, grade, depth, surgical margin, histology) associated with survival outcomes significant at >0.05 were included in the multivariable analysis with the 24-gene signature. Kaplan–Meier survival estimates were produced in GraphPad Prism Version 8.0.2 (San Diego, CA, USA).

The chi-square test was used to compare proportions across categorical factors. The Mann–Whitney *U* test was used to compare median values for continuous variables between two groups. *P* values were two-sided and statistical significance was set as 0.05. When the 24 hypoxia genes were compared individually between hypoxia-low and hypoxia-high tumours the Benjamini (two-stage) method was used to correct for multiple *t* tests with false discovery rate (Q) set at 1%. The likelihood ratio test was used to compare the Cox proportional hazard models of hypoxia in combination with Sarculator versus Sarculator alone.

### Reporting guidelines

Study results are reported according to the Reporting Recommendations for Tumour Marker Prognostic Studies (REMARK) [29].

## Results

### Platform selection for clinical application identifies nanoString for further validation

Thirty-four FFPE biopsy samples were collected prospectively for assay comparison, of which 28 met the minimum RNA concentration required for TLDA (27.8 ng/µl) and nanoString (20 ng/µl). The six samples with low RNA yields contained <20% tumour on pathology review. TLDA data were generated for 26 (93%) and nanoString data for 27 (96%) samples (Supplementary Fig. 1). There were seven (27%, TLDA) and 12 (46%, nanoString) samples classified as hypoxia high. Both assays showed excellent reproducibility with strong correlations (Spearman’s ρ ≥ 0.98) in the expression of the 24 genes in the signature for intra-assay and inter-assay repeats for both low- and high-quality RNA samples (Supplementary Fig. 2). However, there was one discordant hypoxia signature result for an inter-assay repeat for TLDA. For both assays, the reproducibility in measurements of lower expressed genes (high TLDA Ct or low nanoString count) was worse than higher expressed genes in the low-quality sample. This effect was more apparent for the TLDA as some genes were undetermined (Ct = 40). Table 1 summarises factors compared between the two assays. Pass rates for prospective samples (≤3 years old), turnaround times and intensity of labour were similar. The nanoString was superior in terms of reproducibility and pass rates for retrospective samples (10–15 years old) and was taken forward for further clinical validation.

**Table 1:** Platform comparison for targeted assay development for the 24-gene hypoxia signature.

	TLDA	NanoString
Pass rate (prospective)	26/28 (93%)	27/28 (96%)
Pass rate (retrospective)*	10/12 (83%)	280/286 (98%)
Hypoxia high result	7/26 (27%)	12/26 (46%)
RNA input	250 ng, 27.5 ng/µl	100 ng, 20 ng/µl
Intra-assay correlation**	0.99 (low), 1.00 (high)	0.98 (low), 1.00 (high)
Inter-assay correlation***	0.99 (low), 0.99 (high)	0.99 (low), 1.00 (high)
Samples/run	4	12
Hands-on time/run	7 h	6 h
Total time/run	48 h	48 h

*TLDA—pilot study using VORTEX-Biobank samples, NanoString—main validation studies using Manchester and VORTEX-Biobank cohorts.

**Mean Spearman’s ρ for gene expression profiles for triplicate repeats in a single run for a low- and high-quality RNA sample.

***Mean Spearman’s ρ for gene expression profiles for triplicate/quadruplicate repeats across runs for a low- and high-quality RNA sample.

### The 24-gene hypoxia signature nanoString assay detects hypoxia in vitro and in vivo

**Fig. 1:**
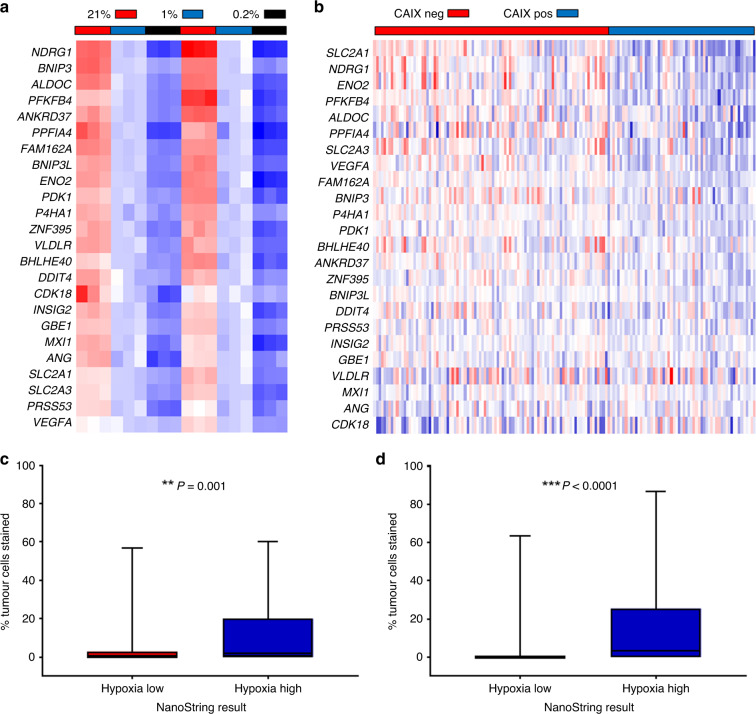
Biological validation of a nanoString assay for the 24-gene hypoxia signature demonstrates that the targeted assay measures hypoxia in vitro and in vivo. Heatmaps of the 24 hypoxia genes (red—low expression, blue – high expression) in (**a**). STS cell lines (HT1080 and SKUT1) cultured under decreasing oxygen concentration (21%, 1%, 0.2%, 24 h of exposure); **b** FFPE tumour samples (CAIX protein expression negative versus positive) from the VORTEX-Biobank (*n* = 152); **c** protein expression of HIF-1α in tumours (*n* = 136) classified as hypoxia-low (red) versus hypoxia high (blue) by the 24-gene signature. **d** Protein expression of CAIX in tumours (*n* = 152) classified as hypoxia-low (red) versus hypoxia high (blue) by the 24-gene signature. Box and whisker plots show the median, interquartile range and range for the percentage of tumour cells stained (immunohistochemistry).

Figure 1 shows the upregulation of the 24 genes in the signature under hypoxia in vitro and in vivo. The nanoString assay was able to detect progressive upregulation of all 24 hypoxia signature genes following exposure of STS cells to decreasing oxygen concentrations (21% versus 1% and 0.2%). All 24 genes were significantly upregulated (21% versus 1% oxygen), whilst the five endogenous control genes were not differentially expressed (Supplementary Table 5).

NanoString data were generated from diagnostic FFPE biopsies for 154 of 216 VORTEX patients (Supplementary Fig. 1). Data were also available for protein expression (IHC) of CAIX (*n* = 152) and HIF-1α (*n* = 136). There were 16/24 hypoxia genes significantly upregulated in CAIX positive versus negative tumours. 10/24 hypoxia genes were significantly upregulated in HIF-1α-positive versus -negative tumours. No endogenous control genes were differentially expressed in tumours expressing either protein hypoxia marker. Higher protein expression of HIF-1α (*P* = 0.0012) and CAIX (*P* < 0.0001) was observed in hypoxia high versus low tumours (Fig. 1).

### The 24-gene hypoxia signature nanoString assay shows low intra-tumour heterogeneity

To assess intra-tumour heterogeneity, multiple FFPE pre-treatment biopsies were collected from 10 patients with a minimum of three samples per tumour (Supplementary Fig. 1). The nanoString hypoxia signature classified 14/45 (31%) samples as hypoxia high. When hypoxia status was determined by the nanoString hypoxia signature, results were concordant in all samples in 9/10 tumours, compared to 4/10 tumours for CAIX protein expression (Supplementary Fig. 3).

### The 24-gene hypoxia signature nanoString assay demonstrates excellent technical performance

**Fig. 2:**
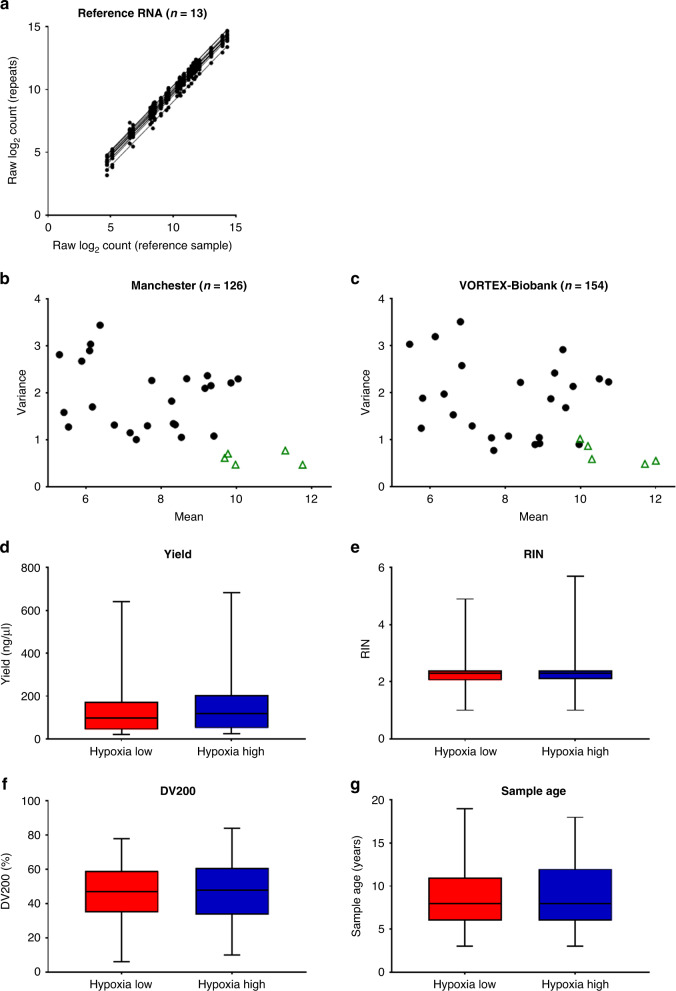
Technical performance of the nanoString assay for the 24-gene hypoxia signature. **a** The raw (pre-normalisation data) gene expression profiles are shown for a single reference RNA sample in the first run versus each subsequent repeat (*n* = 12 repeats). Each data point is the expression value for an individual hypoxia gene. Spearman’s ρ > 0.99 for all repeats. The mean versus variance of the raw expression values for each gene (black data points are hypoxia genes, and green data points are endogenous control genes) are plotted for (**b**). the Manchester cohort; and **c** the VORTEX-Biobank cohort demonstrating low variance of the endogenous controls compared to hypoxia signature genes. RNA quality control measures are shown for hypoxia-low versus hypoxia-high samples from the combined cohort (*n* = 280). No statistically significant differences (Mann–Whitney *U* test) were found in (**d**). RNA yield; **e** RNA integrity number (RIN); **f** DV200 (percentage of RNA fragments over 200 bases) or **g** sample age.

FFPE diagnostic biopsies from two cohorts were used for clinical validation. A nanoString hypoxia signature result was generated for 126/165 (76%) samples from the Manchester cohort and 154/184 (84%) samples from the VORTEX-Biobank cohort (Supplementary Fig. 1). Negative control samples (*n* = 13) showed very little background signal and reference RNA positive control samples demonstrated high reproducibility (*n* = 13, Spearman’s ρ ≥ 0.99) (Fig. 2a). The five endogenous controls performed well in both cohorts, showing low variance compared to the signature genes. They were expressed at a slightly higher level than the hypoxia genes which is expected in endogenous control genes as they are often genes involved in basic cellular processes (Fig. 2b, c).

The nanoString assay classified 53/126 (42%) samples from the Manchester cohort and 70/154 (45%) samples from the VORTEX-Biobank cohort as hypoxia high. There were no significant associations between any RNA quality measures and the nanoString result (Fig. 2d–g). In the Manchester and VORTEX-Biobank cohorts, 10/24 and 17/24 hypoxia genes were significantly upregulated in hypoxia-high tumours, respectively (Supplementary Table 5) in comparison to no endogenous control genes being significantly upregulated in hypoxia-high tumours in either cohort.

### Patients with hypoxia-high tumours have worse clinical outcomes

Table 2 shows the distribution of baseline clinical characteristics by nanoString hypoxia signature result for both cohorts. Hypoxia-high versus low tumours tended to be larger and of a higher grade. A higher proportion of myxofibrosarcoma (56%) and UPS (52%) were classified as hypoxia high compared to myxoid liposarcoma (16%).

**Table 2:** Distribution of baseline clinical characteristics by nanoString hypoxia signature result.

	Manchester cohort, *n* = 126			VORTEX-Biobank cohort, *n* = 154		
	NanoString hypoxia result		*P*	NanoString hypoxia result		*P*
	Low	High		Low	High	
	*n* = 73	*n* = 53		*n* = 84	*n* = 70	
Age (years)	57 (17–86)	63 (23–94)	0.34	58 (23–88)	61 (26–85)	0.06
Size (cm)	8 (2.3–27)	10.3 (2.5–80)	**0.04**	8.7 (1.4–26)	10.0 (2.5–34)	0.06
Sex						
Female	31	21	0.75	36	27	0.54
Male	42	32		48	43	
WHO PS						
0/1	69	44	0.1	68	57	
2/3	2	6		2	1	0.66
Unknown	2	3		16	10	
Surgical margin*						
Intralesional	0	1	0.57			
Marginal	18	16				
Wide	14	10				
Unknown	41	26				
Surgical margin*						
R0				79	60	0.08
R1				5	10	
Depth						
Superficial	14	13	0.47	17	12	0.62
Deep	59	40		67	58	
Grade						
1	10	1	**0.001**	6	2	**0.01**
2	24	15		21	8	
3	28	36		58	59	
Unknown	11	0		–	–	
Histology						
LMS	4	7	0.16	6	1	
MFS	14	7		21	23	
MPNST	8	2		2	3	
MLPS	5	1		15	3	**0.05**
SS	5	3		3	2	
UPS	16	20		22	28	
Other	21	13		15	10	

*WHO PS* World Health Organisation performance status, *MPNST* malignant peripheral nerve sheath tumour, *LMS* leiomyosarcoma, *MLPS* myxoid liposarcoma, *MFS* myxofibrosarcoma, *SS* synovial sarcoma, *UPS* undifferentiated pleomorphic sarcoma.

*P* values are from the Chi^2^ or Mann–Whitney *U* test for categorical and continuous variables, respectively.

*Surgical margins were reported by different systems for the Manchester and VORTEX-Biobank cohorts.

The statistically significant *p* values are shown in bold.

**Fig. 3:**
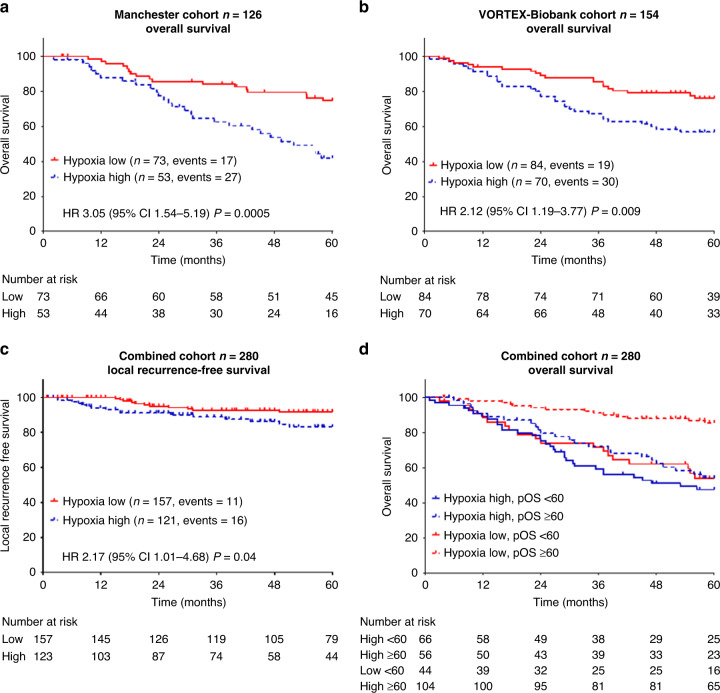
Clinical validation of a 24-gene hypoxia-associated signature in two cohorts when measured in routine pre-treatment FFPE biopsies using a targeted nanoString assay. The graphs show Kaplan–Meier survival estimates in patients with hypoxia-low versus hypoxia-high tumours by the 24-gene nanoString hypoxia assay in the Manchester (*n* = 126), VORTEX-Biobank (*n* = 154) and combined (*n* = 280) cohorts. **a** Overall survival in the Manchester cohort; **b** overall survival in the VORTEX-Biobank cohort; **c** local recurrence-free survival in the combined cohort; **d** overall survival in patients stratified by hypoxia and Sarculator 10-year pOS in the combined cohort. A targeted nanoString assay was used to generate gene expression data from formalin-fixed paraffin-embedded (FFPE) tumour samples. Un-shrunken hypoxia-low and hypoxia-high centroids from the 24-gene STS hypoxia signature in the original training cohort were used to generate hypoxia class predictions. Each new tumour sample was assigned to the nearest class centroid using the Spearman distance. Individual 10-year predicted overall survival was calculated using Sarculator. Patients were classified as clinically high risk (10-year pOS <60%) or clinically low-risk (10-year pOS ≥60%).

In the Manchester cohort, patients with hypoxia-high tumours had worse 5-year MFS (HR 2.04, 95% CI 1.03–4.07, *P* = 0.04), DFS (HR 2.13, 95% CI 1.12–4.05, *P* = 0.02) and OS (HR 3.05, 95% CI 1.54–5.19, *P* = 0.0005) (Fig. 3a), and a non-significant difference in LRFS (HR 3.33, 95% CI 0.86–12.84, *P* = 0.07). The nanoString hypoxia signature result was the only prognostic factor for MFS and DFS and retained prognostic significance in a multivariable analysis for OS (HR 3.39, 95% CI 1.38–5.79, *P* = 0.0012) (Table 3). In the VORTEX-Biobank cohort, patients with hypoxia-high tumours had worse 5-year MFS (HR 1.81, 95% CI 1.10–3.23, *P* = 0.02), DFS (HR 1.89, 95% CI 1.16–3.09, *P* = 0.01) and OS (HR 2.13, 95% CI 1.19–3.77, *P* = 0.009) (Fig. 3b), and a non-significant decrease in LRFS (HR 1.71, 95% CI 0.67–4.35, *P* = 0.18). In a multivariable analysis, no factors retained prognostic significance for MFS, DFS or OS (Table 3).

**Table 3:** Univariate and multivariate survival analyses in the Manchester and VORTEX-Biobank cohorts.

	Manchester cohort, *n* = 126				VORTEX-Biobank cohort, *n* = 154			
	Univariate		Multivariate		Univariate		Multivariate	
	HR (95% CI)	*P*	HR (95% CI)	*P*	HR (95% CI)	*P*	HR (95% CI)	*P*
*Metastasis-free survival*								
Hypoxia	**2**.**04 (1**.**03–4**.**07)**	**0.04**			**1**.**81 (1**.**10–3**.**23)**	**0.02**	1.45 (0.86–2.45)	0.14
Age	0.99 (0.97–1.01)	0.4			1.01 (0.99–1.03)	0.3		
Sex	0.84 (0.42–1.66)	0.6			1.19 (0.71–2.00)	0.5		
WHO PS	0.98 (0.45–2.11)	1			1.02 (0.60–1.73)	0.9		
Size	1.01 (0.99–1.04)	0.3			**1**.**04 (1**.**00–1**.**09)**	**0.03**	1.04 (0.97–1.08)	0.36
Grade	1.81 (0.84–3.90)	0.1			**2.64 (1.26–5.55)**	**0.01**	2.30 (0.82–2.86)	0.18
Depth	2.98 (0.91–9.77)	0.06			1.48 (0.73–3.00)	0.3		
Surgical margin*	3.82 (0.82–17.7)	0.07			1.70 (0.84–3.45)	0.1		
Histology								
LMS	–	–			–	–		
MFS	0.31 (0.06–1.53)	0.15			1.76 (0.41–7.51)	0.45		
MLPS	0.70 (0.12–4.21)	0.7			0.53 (0.088–3.15)	0.48		
MPNST	0.20 (0.02–1.97)	0.17			0.69 (0.06–7.56)	0.76		
SS	0.89 (0.25–3.15)	0.85			2.25 (0.53–9.52)	0.27		
UPS	0.96 (0.19–4.76)	0.96			0.73 (0.066–8.05)	0.8		
Other	0.61 (0.16–2.24)	0.45			1.66 (0.37–7.51)	0.51		
*Disease-free survival*								
Hypoxia	**2.13 (1.12–4.05)**	**0.02**			**1.89 (1.16–3.09)**	**0.01**	1.55 (0.98–2.70)	0.09
Age	1.00 (0.98–1.02)	0.9			1.01 (0.99–1.03)	0.4		
Sex	0.86 (0.45–1.62)	0.6			1.32 (0.79–2.20)	0.3		
WHO PS	1.42 (0.73–2.78)	0.3			1.01 (0.60–1.69)	1		
Size	1.01 (0.98–1.04)	0.5			**1.05 (1.01–1.09)**	**0.02**	1.04 (0.97–2.70)	0.07
Grade	1.81 (0.89–3.66)	0.09			2.12 (1.08–4.17)	0.03	1.79 (0.89–3.60)	0.1
Depth	2.10 (0.79–5.16)	0.1			1.59 (0.79–3.22)	0.2		
Surgical margin*	2.16 (0.68–6.90)	0.2			1.89 (0.93–3.71)	0.06		
Histology								
LMS	–	–			–	–		
MFS	0.38 (0.10–1.43)	0.15			1.87 (0.44–7.98)	0.4		
MLPS	0.51 (0.09–2.81)	0.44			0.52 (0.09–3.14)	0.48		
MPNST	0.31 (0.06–1.71)	0.18			1.57 (0.22–11.1)	0.65		
SS	0.73 (0.24–2.23)	0.57			2.38 (0.56–10.0)	0.24		
UPS	0.70 (0.16–3.13)	0.64			0.74 (0.067–8.11)	0.8		
Other	0.45 (0.14–1.47)	0.19			1.67 (0.37–7.54)	0.5		
*Overall survival*								
Hypoxia	**3.05 (1.54–5.19)**	**0.0005**	**3.39 (1.38–5.79)**	**0.0012**	**2.13 (1.19–3.77)**	**0.009**	1.71 (0.94–3.09)	0.08
Age	1.02 (1.00–1.03)	0.1			1.02 (1.00–1.04)	0.05		
Sex	1.36 (0.73–2.51)	0.3			1.38 (0.76–2.51)	0.3		
WHO PS	1.59 (0.84–3.02)	0.2			1.19 (0.67–2.13)	0.6		
Size	1.02 (0.99–1.04)	0.2			**1.05 (1.01–1.10)**	**0.03**	1.04 (1.00–1.09)	0.08
Grade	**2.07 (1.07–4.01)**	**0.03**	1.46 (0.60–3.20)	0.35	**2.68 (1.14–6.29)**	**0.02**	2.21 (0.92–5.31)	0.08
Depth	1.31 (0.61–2.81)	0.5			1.73 (0.74–4.06)	0.2		
Surgical margin*	1.58 (0.58–4.27)	0.4			2.11 (0.99–4.51)	0.05		
Histology								
LMS	–	–	–	–	–	–		
MFS	0.25 (0.08–0.80)	0.2	0.45 (0.14–1.50)	0.19	2.74 (0.36–20.6)	0.33		
MLPS	0.36 (0.07–1.75)	0.21	1.31 (0.23–7.56)	0.76	0.34 (0.02–5.36)	0.44		
MPNST	0.29 (0.07–1.12)	0.07	0.50 (0.12–2.02)	0.33	1.33 (0.083–21.3)	0.84		
SS	0.59 (0.24–1.43)	0.24	0.57 (0.23–1.43)	0.23	3.15 (0.42–23.5)	0.26		
UPS	0.13 (0.016–1.07)	0.06	0.18 (0.02–1.49)	0.11	1.39 (0.09–22.3)	0.82		
Other	**0.33 (0.13–0.88)**	**0.03**	**0.33 (0.11–0.97)**	**0.045**	3.03 (0.39–23.7)	0.29		

*WHO PS*   World Health Organization performance status, *LMS*   leiomyosarcoma, *MFS*   myxofibrosarcoma, *MLPS*   myxoid liposarcoma, *MPNST*   malignant peripheral nerve sheath tumour, *SS*   synovial sarcoma, *UPS*   undifferentiated pleomorphic sarcoma, *other*   other histology with < 5 cases in total.

*P* values are from the log-rank test.

Age and size were analysed as continuous variables. Hypoxia, sex, WHO PS, grade, depth, surgical margin and histology were analysed as categorical variables. Hypoxia—high versus low; sex—male versus female; WHO PS 1/2 versus 0; Grade—I/II versus III; Depth—deep versus superficial. Histology—each versus LMS.

*For the Manchester cohort marginal versus wide—note missing data for 67/126 (54%) patients; for the VORTEX-Biobank cohort R1 versus R0.

Statistically significant *p* values (<0.05) shown in bold.

When the Manchester and VORTEX-Biobank cohorts were combined, patients with hypoxia-high tumours had worse 5-year LRFS (HR 2.17, 95% CI 1.01–4.68, *P* = 0.04) (Fig. 3c), MFS (HR 1.92, 95% CI 1.28–2.87, *P* = 0.001), DFS (HR 2.00, 95% CI 1.35–2.95, *P* = 0.0004) and OS (HR 2.38, 95% CI 1.57–3.62, *P* = 0.0003). The nanoString hypoxia signature result remained prognostic in a multivariable analysis for MFS (HR 1.71, 95% CI 1.11–2.63, *P* = 0.0014), DFS (HR 1.86, 95% CI 1.24–2.81, *P* = 0.003) and OS (HR 2.24, 95% CI 1.42–3.53, *P* = 0.00096) (Supplementary Table 6). It was the only factor to remain prognostic for OS in the multivariate analysis.

### Prevalence of tumour hypoxia in STS varies by histologic subtype

The classification of tumours by the nanoString hypoxia signature within specific histologic subtypes in the pooled cohorts (*n* = 280) is summarised in Supplementary Fig. 4. Myxoid liposarcoma had the fewest tumours classified as hypoxia high (4/24, 16.7%) and UPS had the highest proportion classified as hypoxia high (48/85, 56.5%). Despite small numbers, the Kaplan–Meier OS curves still appear to separate for hypoxia high versus low tumours within individual subtypes (Supplementary Fig. 4b–e).

### Combining the 24-gene hypoxia signature nanoString assay and the Sarculator nomogram improves prognostication

In the combined cohorts, the Sarculator nomogram 10-year pOS was found to be a good predictor of the observed OS (HR 0.09, 95% CI 0.03–0.23, C-index 0.66, standard error [SE] 0.03). Including the nanoString hypoxia classification improved the fit to the observed survival data (C-index 0.68, SE 0.03, *P* = 0.002). The fit to survival data was further improved when we considered an interaction between hypoxia classification and Sarculator i.e., Sarculator in low hypoxia HR 0.23 (95% CI 0.08–0.68) and in high hypoxia HR 0.06 (95% CI 0.02–0.17), *P* = 0.047. Calibration plots are shown in Supplementary Fig. 5.

In the re-analysis of EORTC-STBSG, a cut-off of ≤60% 10-year pOS was proposed to select patients most likely to benefit from adjuvant chemotherapy. When using this cut-off to define Sarculator high risk in the combined cohort, hypoxia-low/pOS ≥60% of patients had a particularly favourable prognosis compared to other groups. The 5-year OS was 85% for hypoxia [25]-low/pOS ≥60% versus 47–54% for other groups (log-rank *P* = 0.0001) (Fig. 3d).

## Discussion

This study confirmed that the 24-gene hypoxia signature nanoString assay can be used in routine pre-treatment FFPE biopsies alone or in conjunction with clinical risk factors to identify patients with a poor prognosis. Risk stratification based on a tumour microenvironmental feature may be particularly useful in this rare group of tumours, as it is present across many heterogenous histologic subtypes and occurs more frequently than specific genetic driver mutations [30]. The 24-gene hypoxia signature nanoString assay could be used to define a higher-risk population for trials of neoadjuvant/adjuvant systemic therapy. Previous ‘all-comer’ trials have failed to show benefit [5], and a histology-driven approach to neoadjuvant chemotherapy was not successful [31]. The Sarculator nomogram has been proposed as a tool to select patients more likely to benefit from systemic therapy [7]. However, combining Sarculator and the nanoString hypoxia signature improved prognostication compared to either alone and so tailoring treatment based on clinical risk factors and tumour biology may be a useful strategy for future trials.

Another prognostic gene signature, CINSARC [32], based on genome instability has been developed for STS. This has been well validated as a prognostic marker and can also be measured in pre-treatment FFPE biopsies using a nanoString assay [33]. We previously demonstrated that the 24-gene hypoxia signature and CINSARC were independent prognostic factors and that combining them improves prognostication with patients deemed high risk by both having a particularly poor prognosis (5-year MFS ~20%) [25]. Combining measures of genome instability and hypoxia has also been shown to improve prognostication in prostate cancer [34].

In the combined cohort, patients with hypoxia-high tumours had worse LRFS, which was not seen in the individual cohorts due to low numbers of local recurrence events. This effect on local recurrence was not observed when the signature was previously explored in the TCGA cohort (in which only 31% received radiotherapy). The finding may indicate that hypoxic radioresistance in radiotherapy-treated patients increases risk of local recurrence. It is also notable that when hypoxia was explored within individual histologic subtypes, myxoid liposarcomas (which are considered to be clinically radio-responsive) were mostly classified as hypoxia-low. This suggests that their clinical radio-responsiveness may reflect a low burden of hypoxic radioresistance. The 24-gene hypoxia signature nanoString assay could be used to identify patients for trials of radiotherapy + /– hypoxia modification, which may be particularly useful for situations in which a response to neoadjuvant radiotherapy would be useful, such as borderline operable STS or locally recurrent disease.

In addition, the use of multiple biomarkers measuring distinct features of tumour biology will be crucial in directing patients towards appropriate clinical trials of more targeted therapies. Despite showing initial promise, the hypoxia-targeted pro-drug evofosfamide did not demonstrate efficacy in a Phase III trial in metastatic STS [21]. The lack of a hypoxia biomarker was noted as a flaw in the trial. Other hypoxia-targeted therapies have shown benefit specifically in patients with hypoxic tumours, such as nimorazole in head and neck squamous cell carcinoma (HNSCC) [24] and carbogen and nicotinamide (CON) in bladder cancer [35].

The study developed a targeted assay to measure a 24-gene signature as a biomarker of tumour hypoxia in STS for use on routine, pre-treatment FFPE biopsies. Whilst most commercially available gene signature assays use RT-qPCR-based or nanoString technologies, few studies have compared the two. The high pass rates and reproducibility observed with both platforms are consistent with previous reports in FFPE tissue [33, 36]. The nanoString platform was slightly superior in terms of reproducibility and has the important advantage that it can measure many more genes from a single sample simultaneously and so has more scope to combine measuring hypoxia with other biological signatures in the future. It is important to note that the 24-gene signature is platform agnostic, having been validated previously in microarray and RNA-Seq data [25] and in nanoString data in the current cohorts. Whole genome sequencing is becoming more routine and so the ability to transfer to sequencing-based platforms using common equipment already in place in clinical laboratories will be useful for future implementation in clinical trials.

In the assay development (MCRC-Biobank) and validation (Manchester and VORTEX-Biobank) cohorts, the nanoString assay demonstrated excellent technical performance with a high degree of intra-assay and inter-assay reproducibility and low turnaround times which suggests it could be used effectively in a prospective clinical trial. It was successful in detecting hypoxia in vitro and correlated with other specific protein markers of tumour hypoxia. This biological validation step is vital if the biomarker is to be used to select patients for trials of hypoxia-targeted therapies, as many gene signatures can be prognostic without specific biological relevance [37]. Another hypoxia signature developed for head and neck cancer has been reported to be prognostic in STS in a smaller cohort, however, that signature result did not correlate with other measures of hypoxia (direct electrode measurements) [38].

The 24-gene hypoxia signature nanoString assay result showed considerably less intra-tumour heterogeneity than a single protein marker. This is consistent with previous studies demonstrating that larger multi-gene biological signatures are more tolerant of intra-tumour heterogeneity than smaller signatures or single markers, possibly as the result is less reliant on the expression of a single hypoxia marker across multiple heterogenous tumours [39, 40]. The advantage of this is that a single pre-treatment biopsy would likely be sufficient in a prospective clinical trial.

This is the largest study to date of a tumour microenvironment classifier in STS; however, some limitations of the work should be recognised. Due to the rarity of STS the cohorts are relatively small and underpowered for multivariable analyses, which was partly overcome by pooling data in the combined cohort. For the Manchester cohort, data and tissue collection were retrospective. In the combined cohort it was not possible to include a surgical margin in the LRFS multivariable analyses due to the use of different reporting systems, which should be standardised in future trials. Both cohorts were treated with adjuvant radiotherapy, however, standard practice is moving more towards using neoadjuvant radiotherapy and further investigation of the signature with the assessment of radiation response in the surgical specimens in a cohort treated with neoadjuvant radiotherapy is an important future aim. Finally, the cohorts consisted exclusively of limb STS and it would be useful to consider the impact of hypoxia on radiation response and local recurrence at other anatomical sites, such as retroperitoneal STS.

In summary, we have validated a 24-gene signature as a biomarker of tumour hypoxia in STS. The signature has undergone robust biological validation and in previous [25], and in the current work its prognostic value for MFS and OS has now been validated in over 800 patients across four independent cohorts, including a Phase III clinical trial. The nanoString assay demonstrated excellent technical performance and was able to reliably measure the hypoxia signature on diagnostic pre-treatment FFPE biopsies. The signature measures hypoxia across multiple STS subtypes. Potential future uses of the hypoxia signature in prospective clinical trials include the selection of patients: (1) with poor prognosis at high risk of metastasis for clinical trials of neoadjuvant/adjuvant chemotherapy; (2) who may benefit from the addition of hypoxia modification to radiotherapy; and (3) for biomarker-driven trials of systemic hypoxia targeted therapy.

## Supplementary information

Supplementary Methods

Supplementary Figures

Supplementary Tables

## Data Availability

Gene expression data (nanoString) for the Manchester and VORTEX-Biobank cohorts will be made publicly available via the Gene Expression Omnibus (GEO) immediately following publication with no end date. The corresponding clinical data for the Manchester cohort can be accessed following approval by the lead responsible clinician (JW). Clinical data for VORTEX (de-identified individual participant data collected during the main VORTEX trial) can be accessed by investigators whose proposed use of the data has been approved by the VORTEX Chief Investigator (MR) and the VORTEX-Biobank Translational Lead (CW).
