# Supplementary material for: Technical development and validation of a clinically applicable microenvironment classifier as a biomarker of tumour hypoxia for soft tissue sarcoma
Source: Br J Cancer. 2023 Apr 21;128(12):2307–17. doi: 10.1038/s41416-023-02265-3 (PMC10241814; doi:10.1038/s41416-023-02265-3)
Supplement: Supplementary file 1 — Supplementary Methods [file 41416_2023_2265_MOESM1_ESM.docx]

# Supplementary methods

## Clinical cohorts

### Manchester Cancer Research Centre (MCRC) biobank cohort

A recent (≤3 year old) cohort of patients with available formalin-fixed paraffin-embedded (FFPE) STS tumour samples was required for technical studies and prospective assay development. An application was approved to use FFPE tumour samples (n=34) by the MCRC biobank (18/NW/0092). As the samples were required only for assay development work there were no inclusion/exclusion criteria based on histology and clinical outcome data were not required.

### Manchester and intra-tumour heterogeneity cohorts

Patients with extremity STS treated at the Christie NHS Foundation Trust were identified through a clinical database and FFPE tumour tissue requested from the original biopsy from the referring hospital (n=165). An additional cohort (n=10) with multiple available FFPE tumour samples (3-8 per patient) was selected from the database for intra-tumour heterogeneity studies. The study had ethical approval (06Q1403256). The samples were not identifiable to the research team so informed consent was not required.

Patients were diagnosed between 1990 and 2015; median follow up was 65 months and median age at diagnosis was 60 years. Baseline data were collected for the database at the first clinic appointment and follow up data entered at each subsequent appointment. Baseline and outcome data were extracted from the database for all patients in March 2018. The 5-year event rates for local recurrence, metastasis, any recurrence and death were 6%, 22%, 26% and 29%, respectively.

### VORTEX-Biobank cohort

VORTEX was a randomised, controlled, multi-centre, phase III clinical trial comparing two radiotherapy volumes in adults with extremity STS treated radically with surgery and post-operative radiotherapy. Inclusion criteria were age 16 years or over, histologically proven STS originating in the extremity and no prior radiotherapy to the local site. If a patient had undergone excisional biopsy with positive margins, they were only eligible following re-excision. Patients with a previous invasive malignancy were eligible if they had been disease free for over three years. Exclusion criteria included patients with rhabdomyosarcoma (alveolar or embryonal), primitive neuro-ectodermal tumour, soft tissue Ewing’s sarcoma, extraskeletal osteosarcoma, desmoid tumours, dermatofibrosarcoma protuberans, Gorlin’s syndrome, local recurrence of previously treated STS, local recurrence more than three months after previous surgery, concurrent malignancy, regional nodal disease, unequivocal distant metastasis and use of (neo)adjuvant chemotherapy. Pregnant and breast feeding women were excluded.

Patients were registered and gave written informed consent for the VORTEX-Biobank pre-operatively then those eligible for adjuvant radiotherapy were randomised in a 1:1 ratio (stratified by surgical margin, tumour grade and treatment centre) between two treatment arms following surgical resection. Randomisation was performed centrally by the Cancer Research UK Clinical Trials Unit at the University of Birmingham. Trial treatment commenced within 12 weeks of surgery. Clinical outcome data were only available for randomised patients.

Surgery consisted of complete resection of the tumour and an overlying margin of normal tissue unless the tumour contacted a major nerve vessel or bone (when the critical structure was preserved unless there was gross tumour involvement). A central review of histopathology reports and specimens was performed by members of the Trial Management Group and Data Monitoring Committee. Radiotherapy planning was performed individually for each patient. 2D and 3D planning techniques were permitted. The control arm consisted of 50 Gy in 25 fractions to CTV1 (GTV + 5 cm cranio-caudally and 2 cm axially) followed by 16 Gy in 8 fractions to CTV2 (GTV + 2 cm cranio-caudally and axially) and the research arm of 66 Gy in 33 fractions to CTV2 alone. Quality Assurance was performed to ensure the prescribed dose was delivered according to protocol.

Patients underwent pre-operative magnetic resonance imaging (MRI) and then local recurrence was assessed using repeat MRI annually for 2 years and then clinical evaluation. The Toronto Extremity Salvage Score (TESS) and patient perceived change of status questionnaires were performed pre- and post-operatively and then at 3-6 monthly intervals after surgery. The RTOG/EORTC guidelines were used to assess late radiation morbidity of the skin, subcutaneous tissues, bones and joints performed at 3 monthly intervals post-operatively, and then 6-monthly for five years.

The primary outcome measures were (1) limb functionality measured by TESS; and (2) time to local recurrence (time from randomisation to biopsy confirmed local recurrence in whole days).

Secondary outcome measures were (1) RTOG/EORTC soft tissue and bone toxicity; (2) disease-free survival time defined as tiem from randomisation to local recurrence, distant recurrence or death (whichever occurs first); (3) overall survival time defined as time from randomisation to death; and (4) overall level of disability according to TESS.

There was no difference in limb function at 2 years between the control and research arms. Due to the low event numbers for local recurrence it was not possible to draw conclusions as to non-inferiority of the research arm with regards to local recurrence^1^. The 5-year event rates for local recurrence, metastasis, any recurrence and death were 11%, 38%, 40% and 29%, respectively.

Samples collected for the VORTEX-Biobank included the diagnostic FFPE biopsy, tumour and normal tissue frozen in RNA later at the time of surgery and a peripheral blood sample. The study had ethical approval (06/MRE03/3) and written informed consent for sample collection and analysis was obtained.

## Targeted assays

## TaqMan Array Cards (TLDA)

TLDA is a real-time quantitative PCR (RT-qPCR) based technology used to measure gene expression. Custom 384 well microfluidic TaqMan low density array (TLDA) cards (Life Technologies, Paisley, UK) with each well containing a single TaqMan assay were designed for the genes of interest. Pre-designed assays were selected from the Thermofisher website for each gene. Each card measured 32 genes in triplicate for four samples. This included 24 hypoxia signature genes, 1 manufacturing control (*18S*) and 7 endogenous control genes.

For each sample 4 µl of cDNA from reverse transcription was added to 4 µl TaqMan Custom Pre Amp Pool and 8 µl TaqMan PreAmp Master Mix (4391128, Applied Biosystems) to give a total reaction volume of 16 µl. Reactions were cycled on a PCR thermal cycler (Veriti 9902, Life Technologies, UK) at 95°C for 10 min, followed by 14 cycles of 95°C for 15 s and 60°C for 4 min, then 99.9°C for 10 min before reducing to 4°C.

7µl of pre-amplified sample was added to 103 µl of nuclease free water and 110 µl of TaqMan Fast Advanced Master Mix (4444557, Applied Biosystems) resulting in a total volume of 220 µl. Samples were loaded into two adjacent loading wells on the TLDA card (100 µl per loading well). Samples were distributed across wells by centrifuging twice at 300 G for one minute and cards were sealed with a sealing tool (Applied Biosystems). Cards were then placed on a TaqMan Array Card block and quantitative PCR performed on the QuantStudio 12K Flex Real-Time PCR System (Life Technologies) to determine the Ct value as defined above. All experiments used the standard thermal protocol for fast Master Mix (92°C for 10 min, then 40 cycles of 95°C for 1 second and 60°C for 20 s). Three cards were prepared simultaneously with each card containing three samples and one of the controls or reference RNA from each reverse transcription batch of 12.

Raw Ct values for each well were exported from the Thermofisher cloud (Thermo Fisher Scientific). The mean and standard deviation for each gene was calculated. If the standard deviation was >1.0 the furthest outlier was excluded and the mean re-calculated. If the raw Ct was >32 for any endogenous control gene or the raw Ct was >32 for >5 hypoxia genes the sample was considered a fail. For passed samples any genes that were ‘undetermined’ were assigned a Ct value of 40. The geomean of the endogenous controls was calculated and subtracted from the raw Ct of each hypoxia gene (ΔCt). Gene expression values were taken as 2^- ΔCt^.

The maximum amount of RNA (without pre-amplification) that can be input to a TLDA card is 2000 ng. For two samples run with maximum RNA input 11/32 and 14/32 genes failed, demonstrating that for degraded FFPE RNA the pre-amplification step is required. Minimal difference in the normalised expression profiles for the 24-gene signature was seen when a single reference RNA sample was run with and without pre-amplification (data not shown).

RT-qPCR efficiency is defined as the ratio of the target gene molecules at the end of a cycle to those at the beginning of the cycle and ideally should be 95-100%. Reaction efficiencies (e) are calculated as e=10^-1/slope^ when raw Ct values for each gene are plotted across a range of cDNA input amounts. This was performed (as recommended by the manufacturer) for the TLDA cards using a single reference RNA sample and demonstrated linear reaction efficiencies for each individual gene (data not shown).

## NanoString

NanoString is an alternative technology to RT-qPCR used to measure gene expression. A nanoString codeset includes probes for each gene of interest, six positive controls (at fixed concentrations) and eight negative controls (with no target). Codesets were designed by NanoString Technologies (NanoString Technologies, Seattle, WA, US) to include the 24-gene hypoxia signature and seven candidate endogenous control genes. Samples were prepared from stock RNA to give a total of 10 µl at a concentration of 20 ng/µl. Each reaction had a total volume of 15 µl (5 µl RNA sample, 3 µl reporter codeset, 5 µl hybridization buffer and 2 µl capture probeset). Experiments were performed in batches of 12 samples in 12 tube PCR hybridization strips. Two control samples (1 nuclease free water and 1 reference RNA) were included in alternate batches. Samples were incubated for 24 h at 65°C.

Post hybridization sample, wash reagents and imaging cartridges were processed on the nCounter Prep Station (NanoString Technologies) and imaged (maximum resolution 555 fields of view (FOV)) on the nCounter Digital Analyzer (NanoString Technologies) according to the manufacturer’s protocol.

NanoString data were processed using nSolver analysis software 4.0 (NanoString Technologies). The nSolver QC parameters were set as percent FOV registration less than 75% (imaging), binding density 0.05-2.25 (binding density) and positive control R^2^ less than 0.95 (positive control linearity). Background thresholding was not performed due to the relatively low level of background observed. The raw data were used to exclude samples if the counts for any endogenous control or >5 hypoxia genes were lower than the average negative control count for that sample. Positive control normalisation was then performed in nSolver 4.0 (NanoString Technologies). This involves calculating the geomean of positive controls A-E for each sample. A scaling factor is calculated for each sample (cohort average geomean/sample geomean) and the raw count for each gene is multiplied by this. Codeset content normalisation was performed in nSolver using the same process but using the geomean of the endogenous control genes. Gene expression values were taken as the log_2_ transformed normalised counts.

## Targeted assay comparison

TLDA and nanoString assays measuring the 24-gene signature were compared in FFPE tumour samples from the MCRC biobank cohort. These samples were all less than three years old. Intra-assay and inter-assay reproducibility of gene expression profiles and signature results were determined in a single high quality (DV200=75, 18S Ct=26.8) and a single low quality (DV200=30, 18S Ct=32.7) quality RNA sample. For TLDA the samples were run in triplicate on a single card and across three cards and for nanoString the samples were run in triplicate in a single batch and across the four batches.

**Supplementary references**

1. Robinson MH, Gaunt P, Grimer R, et al. Vortex Trial: A Randomized Controlled Multicenter Phase 3 Trial of Volume of Postoperative Radiation Therapy Given to Adult Patients With Extremity Soft Tissue Sarcoma (STS). International Journal of Radiation Oncology • Biology • Physics 2016; 96(2): S1.

2. Forker L, Gaunt P, Sioletic S, et al. The hypoxia marker CAIX is prognostic in the UK phase III VorteX-Biobank cohort: an important resource for translational research in soft tissue sarcoma. Br J Cancer 2018; 118(5): 698-704.

3. Bolger AM, Lohse M, Usadel B. Trimmomatic: a flexible trimmer for Illumina sequence data. Bioinformatics 2014; 30(15): 2114-20.

4. Dobin A, Davis CA, Schlesinger F, et al. STAR: ultrafast universal RNA-seq aligner. Bioinformatics 2013; 29(1): 15-21.

5. Love MI, Huber W, Anders S. Moderated estimation of fold change and dispersion for RNA-seq data with DESeq2. Genome Biol 2014; 15(12): 550-.

6. Vandesompele J, De Preter K, Pattyn F, et al. Accurate normalization of real-time quantitative RT-PCR data by geometric averaging of multiple internal control genes. Genome Biol 2002; 3(7): research0034.1.

7. Yang L, Forker L, Irlam JJ, et al: Validation of a hypoxia related gene signature in multiple soft tissue sarcoma cohorts. Oncotarget 9:3946-3955, 2017

8. Hastie T, Tibshirani R, Narasimhan B, Chu G. pamr: Pam: Prediction Analysis for Microarrays. 2019. https://CRAN.R-project.org/package=pamr.
