# Supplementary material for: Technical development and validation of a clinically applicable microenvironment classifier as a biomarker of tumour hypoxia for soft tissue sarcoma
Source: Br J Cancer. 2023 Apr 21;128(12):2307–17. doi: 10.1038/s41416-023-02265-3 (PMC10241814; doi:10.1038/s41416-023-02265-3)
Supplement: Supplementary file 2 — Supplementary Figures [file 41416_2023_2265_MOESM2_ESM.docx]

**
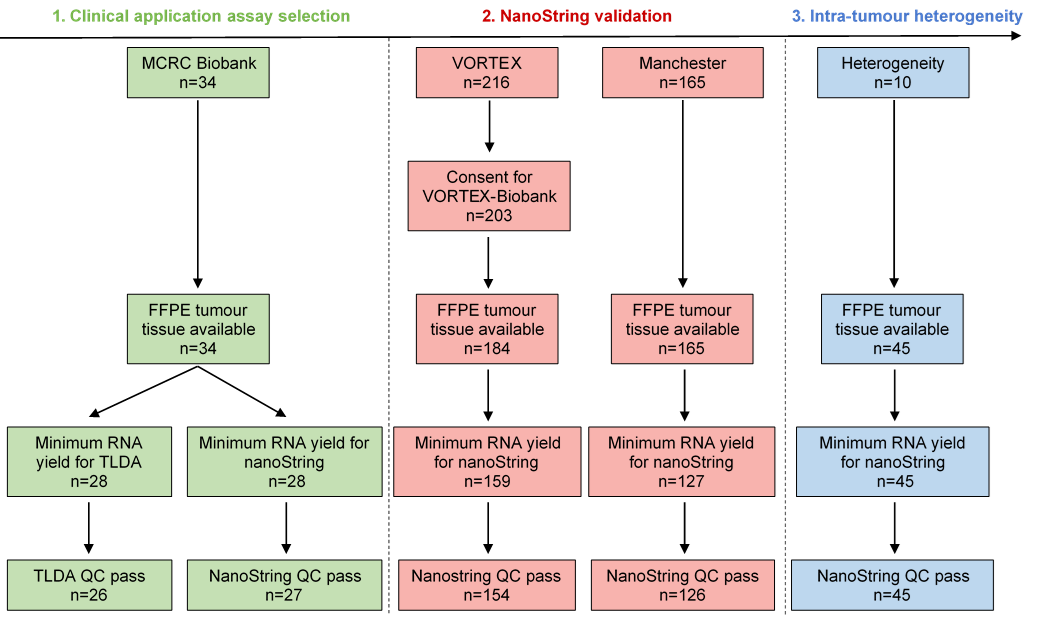
**

**Supplementary Figure 1. Study design and CONSORT diagram.** A summary of patients, available samples and study design for 24-gene hypoxia signature clinical application targeted assay selection, validation and intra-tumour heterogeneity studies is shown. Minimum RNA yield for TLDA and nanoString were 27.8 ng/µl and 20 ng/µl, respectively. Quality control parameters are detailed in the supplementary methods.


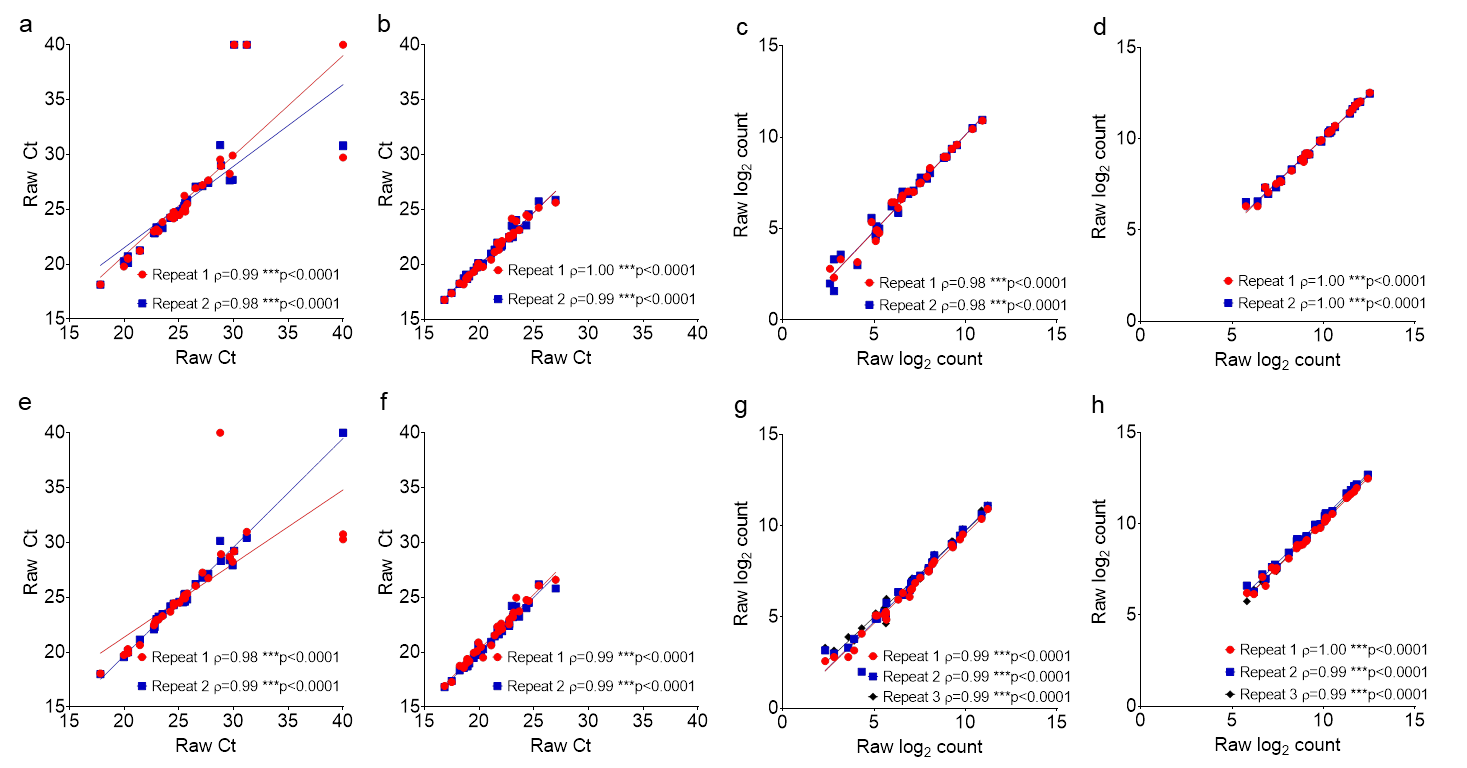


#### Supplementary figure 2. Intra-assay and inter-assay reproducibility of TLDA versus nanoString assays. Raw (pre-normalisation data) gene expression profiles for a single FFPE derived RNA sample in the first run versus each subsequent repeat. Each data point is the expression value for an individual hypoxia gene. Spearman’s ρ and the p-value are shown for each repeat. a. intra-assay reproducibility of TLDA in a low-quality sample; b. intra-assay reproducibility of TLDA in a high-quality sample; c. intra-assay reproducibility of nanoString in a low-quality sample; d. intra-assay reproducibility of nanoString in a high-quality sample; e. inter-assay reproducibility of TLDA in a low quality sample; f. inter-assay reproducibility of TLDA in a high quality sample. g. inter-assay reproducibility of nanoString in a low-quality sample. h. inter-assay reproducibility of nanoString in a high-quality sample.


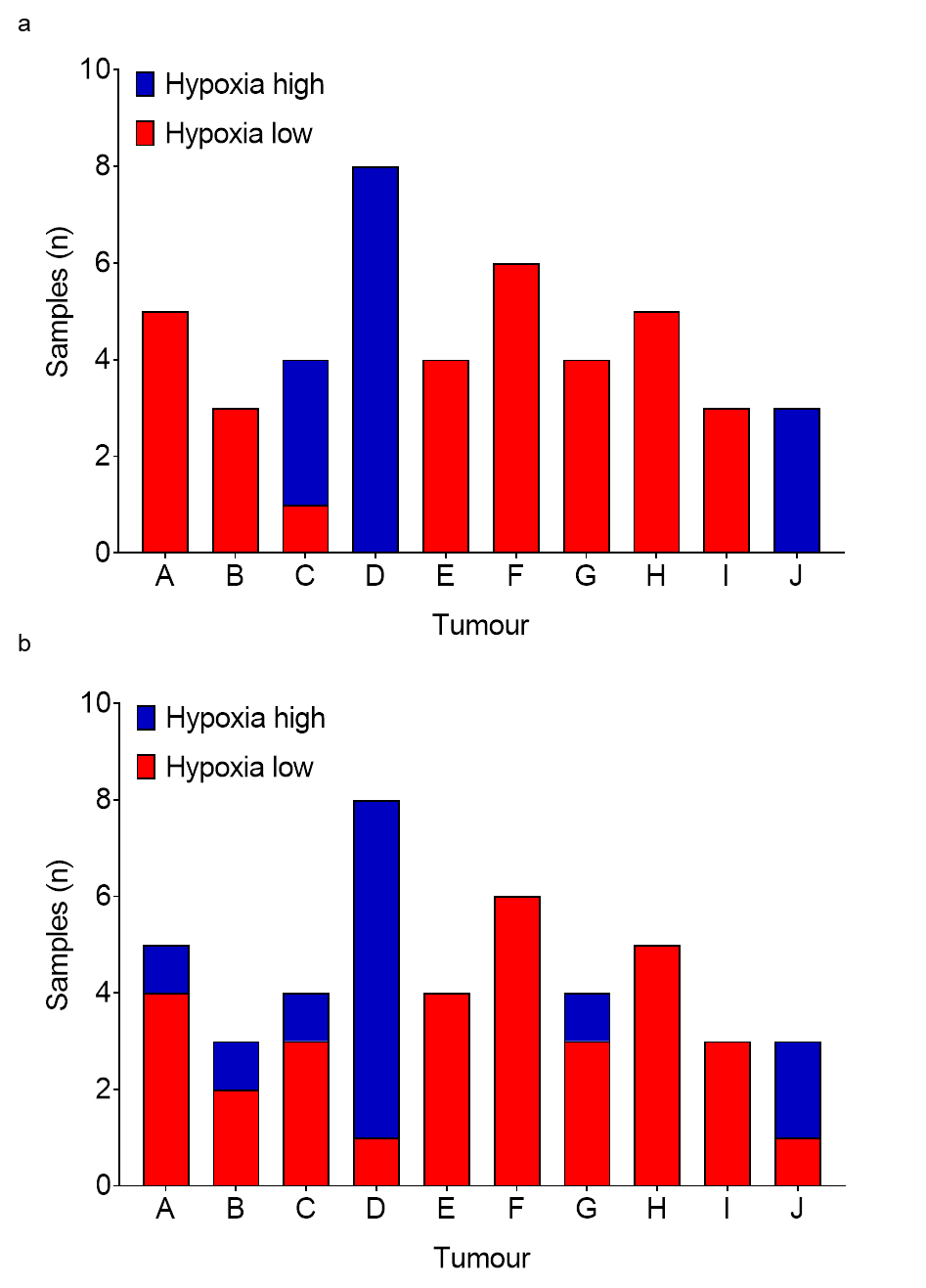


#### Supplementary figure 3. Intra-tumour heterogeneity of nanoString hypoxia signature result versus CAIX protein expression. Intra-tumour heterogeneity in multiple FFPE samples (3-8 per tumour) from 10 tumours (A-J). Each bar shows the number of samples classified as hypoxia-low (red) or hypoxia-high (blue) by the 24-genehypoxia signature (nanoString assay) (a) and CAIX protein expression (immunohistochemistry) (b).


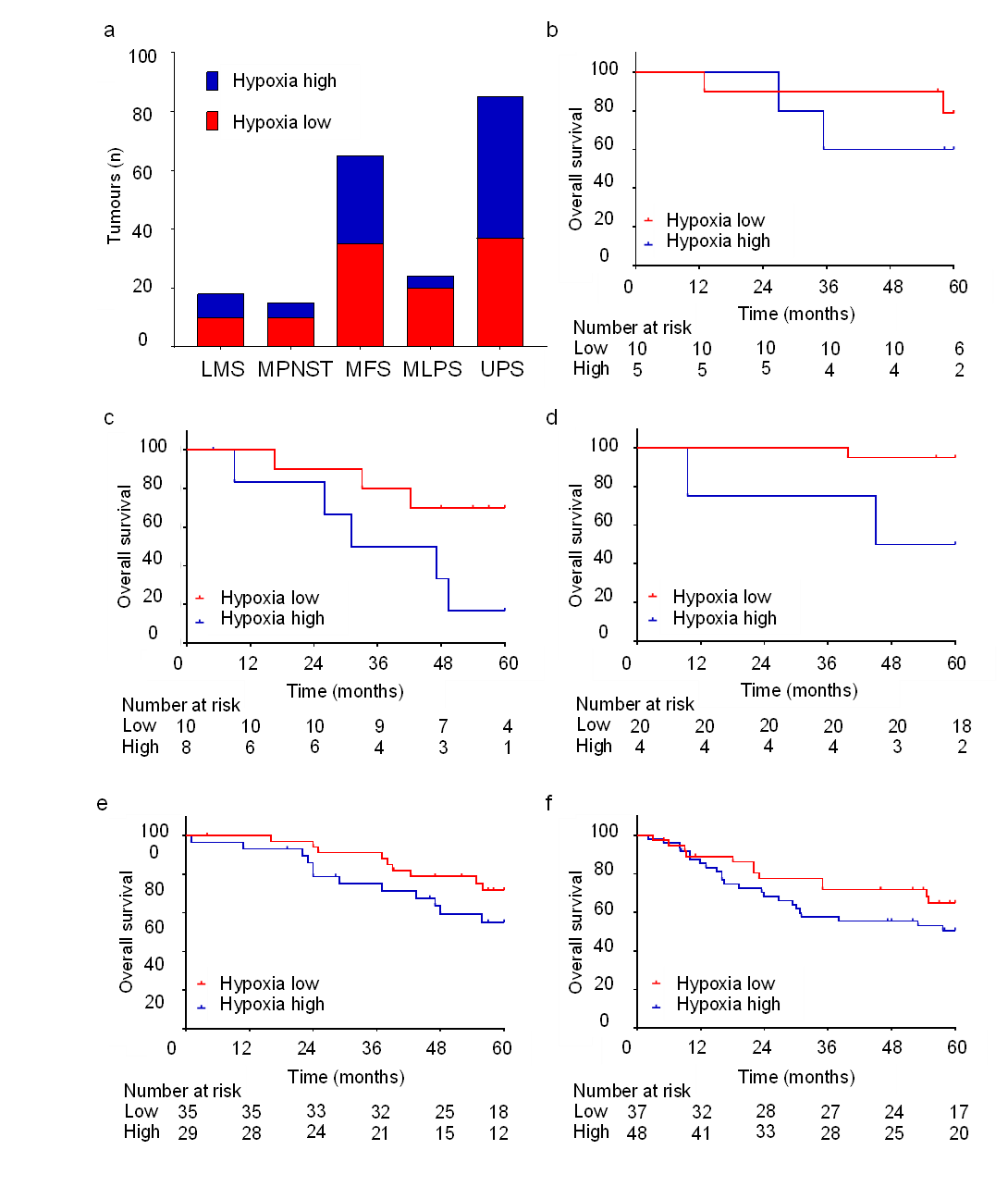
 **Supplementary figure 4. 24-gene hypoxia signature results by histologic subtype in the combined cohorts**. a. The number of tumours classified by the nanoString hypoxia signature as hypoxia-low and hypoxia-high within subtypes with ≥15 cases is shown. Kaplan-Meier survival estimates for OS for patients with hypoxia-low versus hypoxia-high tumours in b. MPNST (n=15); c. LMS (n=18); d. MLPS (n=24); e. MFS (n=65); and d. UPS (n=85). MPNST = malignant peripheral nerve sheath tumour, LMS = leiomyosarcoma, MLPS = myxoid liposarcoma, MFS = myxofibrosarcoma, UPS = undifferentiated pleomorphic sarcoma.


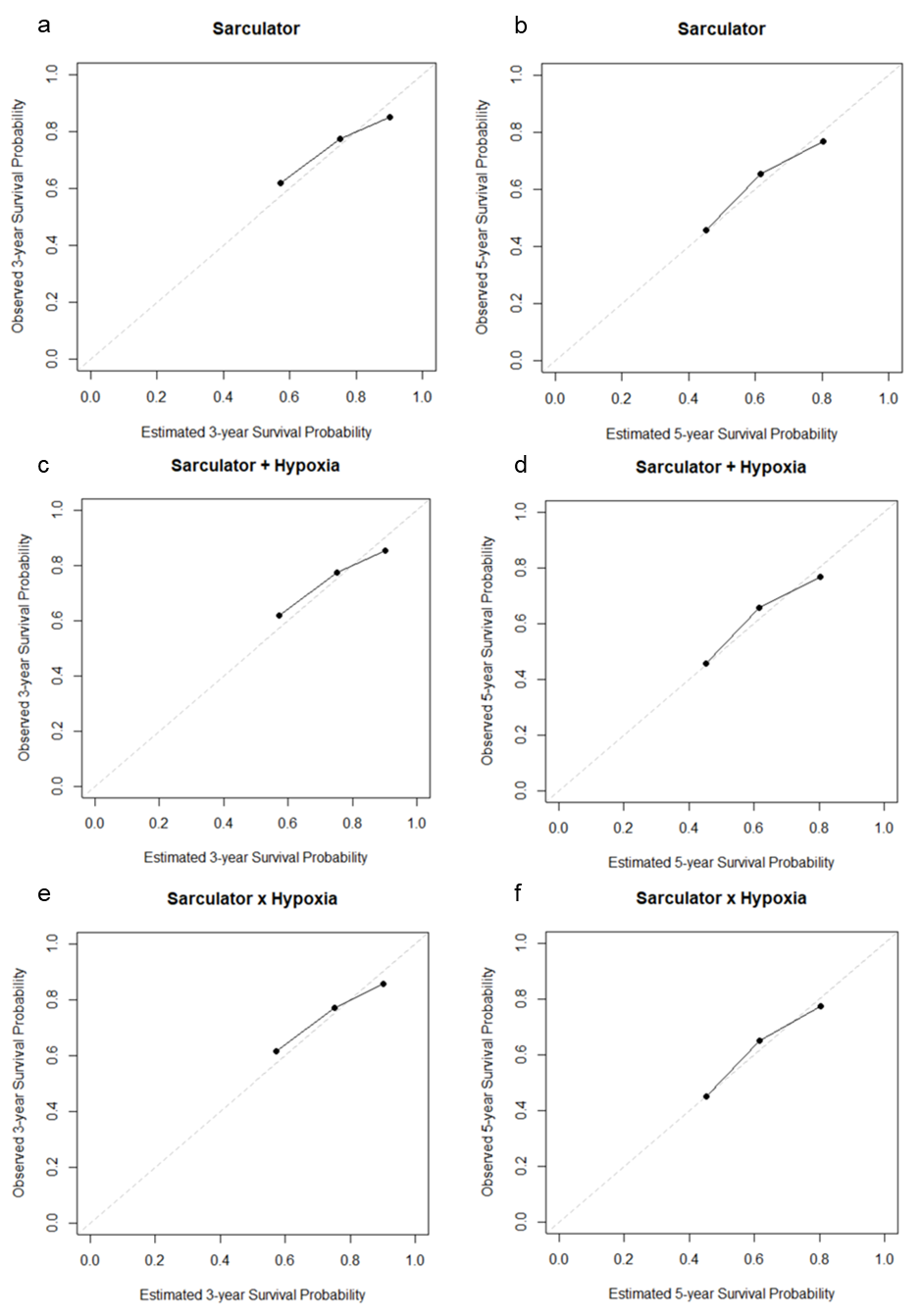


**Supplementary figure 5. Calibration plots for Sarculator nomogram and nanoString hypoxia signature**. a & b. Sarculator, c & d. Sarculator + Hypoxia and e & f. Sarculator interaction with hypoxia at 3 and 5 years.
