# Supplementary material for: Technical development and validation of a clinically applicable microenvironment classifier as a biomarker of tumour hypoxia for soft tissue sarcoma
Source: Br J Cancer. 2023 Apr 21;128(12):2307–17. doi: 10.1038/s41416-023-02265-3 (PMC10241814; doi:10.1038/s41416-023-02265-3)
Supplement: Supplementary file 3 — Supplementary Tables [file 41416_2023_2265_MOESM3_ESM.docx]

#### Supplementary table 1. MCRC biobank cohort samples.

| **Sample** | **Site** | **Histology** | **% Tumour** | **Yield* (ng/µl)** | **260/280** | **260/230** | **RIN** | **DV200** |  |
| --- | --- | --- | --- | --- | --- | --- | --- | --- | --- |
| 1 | retroperitoneal | DDLPS | 50-60 | 81 | 1.79 | 1.10 | 2.4 | 61 |  |
| 2 | retroperitoneal | DDLPS | 60 | 140.7 | 1.89 | 1.50 | 2.2 | 56 |  |
| 3 | retroperitoneal | DDLPS | 45-50 | 291.6 | 1.92 | 1.79 | 2.6 | 30 |  |
| 4 | retroperitoneal | DDLPS | 70 | 72 | 1.83 | 1.32 | 2.4 | 55 |  |
| 5 | lung | ULMS | 60 | 84 | 1.90 | 1.50 | 1.6 | 61 |  |
| 6 | retroperitoneal | DDLPS | 60 | 84.8 | 1.83 | 1.14 | 2.4 | 65 |  |
| 7 | lung | SS | 85-90 | 106.6 | 1.96 | 1.92 | 2.4 | 71 |  |
| 8 | retroperitoneal | LPS | <10 | 7.61 | 1.60 | 0.70 | n/a | n/a |  |
| 9 | retroperitoneal | DDLPS | >90 | 33.8 | 1.75 | 1.05 | 2.1 | 54 |  |
| 10 | chest wall | MFS | 90 | 50.6 | 1.91 | 1.73 | 2.0 | 66 |  |
| 11 | lung | UPS | 70 | 421.2 | 1.98 | 1.91 | 2.4 | 70 |  |
| 12 | uterine | LMS | 80 | 122.4 | 1.95 | 1.80 | 2.6 | 67 |  |
| 13 | retroperitoneal | LPS | 10 | 4.46 | 1.60 | 0.40 | 2.3 | 56 |  |
| 14 | prostate | LMS | 70 | 421.2 | 2.00 | 1.95 | 2.1 | 46 |  |
| 15 | lower limb | MFS | 15 | 215.1 | 1.95 | 1.58 | 2.2 | 51 |  |
| 16 | abdomen | WDLPS | <10 | 5.31 | 1.73 | 1.22 | 2.2 | 53 |  |
| 17 | retroperitoneal | LMS | 65 | 204.9 | 1.99 | 1.86 | 2.3 | 32 |  |
| 18 | stomach | LMS | >90 | 172.8 | 1.94 | 1.93 | 2.6 | 75 |  |
| 19 | lung | UPS | 75 | 31.9 | 1.66 | 0.66 | 2.2 | 46 |  |
| 20 | chest wall | UPS | 20 | 22 | 1.85 | 1.25 | 2.0 | 70 |  |
| 21 | unknown | DDLPS | 60 | 110.6 | 1.92 | 0.64 | 2.3 | 40 |  |
| 22 | unknown | LMS | 33 | 62.6 | 1.87 | 1.19 | 2.3 | 74 |  |
| 23 | retroperitoneal | DDLPS | 70 | 38.6 | 1.70 | 0.64 | 2.4 | 30 |  |
| 24 | retroperitoneal | DDLPS | 60 | 207.3 | 1.94 | 1.78 | 2.4 | 17 |  |
| 25 | abdominal wall | UPS | 30-40 | 60.2 | 1.75 | 0.90 | 2.3 | 55 |  |
| 26 | lung | ULMS | 95 | 247.6 | 2.02 | 1.97 | 2.1 | 57 |  |
| 27 | lung | MFS | 95 | 62.1 | 1.99 | 1.94 | 2.4 | 43 |  |
| 28 | retroperitoneal | LPS | <10 | 7.39 | 1.85 | 1.19 | 2.1 | 50 |  |
| 29 | abdominal wall | UPS | <10 | 4.2 | 1.58 | 0.29 | 2.5 | 50 |  |
| 30 | thigh | LMS | 60 | 27.4 | 1.83 | 0.13 | 2.4 | 45 |  |
| 31 | retroperitoneal | DDLPS | 50 | 54.9 | 1.94 | 0.18 | 2.1 | 57 |  |
| 32 | unknown | UPS | 65 | 436.2 | 2.00 | 2.05 | 2.2 | 36 |  |
| 33 | lung | unknown | 65 | 200.1 | 1.98 | 1.73 | 2.2 | 67 |  |
| 34 | abdomen | UMLS | n/a** | 38.3 | 1.75 | 0.68 | 2.6 | 11 |  |
| **Mean** |  |  |  | **125**.**1** |  |  | **2**.**3** | **52** |  |
| **Qubit yield*  **** *H+E unavailable*  *DDLPS = de-differentiated liposarcoma; ULMS = uterine leiomyosarcoma; SS = synovial sarcoma; LPS = liposarcoma; MFS = myxofibrosarcoma; UPS = undifferentiated pleomorphic sarcoma; LMS = leiomyosarcoma; WDLPS = well-differentiated liposarcoma* | | | | | | | | | |

#### Supplementary table 2. Baseline clinical features for the Manchester and VORTEX-Biobank cohorts.

|  | **Manchester** | | **VORTEX-Biobank** | |
| --- | --- | --- | --- | --- |
|  | **n=165** | **%** | **n=203** | **%** |
| **Sex** |  |  |  |  |
| **Female** | 67 | 40.6 | 82 | 40.4 |
| **Male** | 98 | 59.4 | 121 | 59.6 |
| **WHO PS** |  |  |  |  |
| **0** | 111 | 67.3 | 121 | 63.5 |
| **1** | 32 | 19.4 | 34 | 16.7 |
| **2/3** | 14 | 8.5 | 5 | 2.5 |
| **unknown** | 8 | 4.8 | 35 | 17.2 |
| **Grade** |  |  |  |  |
| **1** | 24 | 14.5 | 14 | 7.9 |
| **2** | 49 | 29.7 | 39 | 19.2 |
| **3** | 77 | 46.7 | 150 | 73.9 |
| **unknown** | 15 | 9.1 | 0 | 0.0 |
| **Size (median [range] cm)** | 8.0 (0.5 – 27.0) | | 9.0 (1.4 - 34.0) | |
| **Depth** |  |  |  |  |
| **superficial** | 37 | 22.4 | 36 | 17.7 |
| **deep** | 126 | 76.4 | 166 | 81.8 |
| **unknown** | 2 | 1.2 | 1 | 0.5 |
| **Radiotherapy** |  |  |  |  |
| **yes** | 108 | 65.5 | 203 | 100 |
| **no** | 16 | 9.7 | 0 | 0.0 |
| **unknown** | 41 | 24.8 | 0 | 0.0 |
| **Chemotherapy** |  |  |  |  |
| **yes** | 5 | 3.0 | 0 | 0.0 |
| **no** | 119 | 72.1 | 203 | 100 |
| **unknown** | 41 | 24.8 | 0 | 0.0 |
| **Surgical margins*** |  |  |  |  |
| **intralesional** | 4 | 2.4 |  |  |
| **marginal** | 50 | 30.3 |  |  |
| **wide** | 37 | 22.4 |  |  |
| **radical** | 0 | 0.0 |  |  |
| **unknown** | 74 | 44.8 |  |  |
| **Surgical margins*** |  |  |  |  |
| **R0** |  |  | 182 | 89.7 |
| **R1** |  |  | 21 | 10.3 |
| **Histology** |  |  |  |  |
| **DDLPS** | 5 | 3.0 | 5 | 2.5 |
| **ECMS** | 5 | 3.0 | 0 | 0.0 |
| **LMS** | 15 | 9.1 | 9 | 4.4 |
| **LPS** | 10 | 6.0 | 0 | 0.0 |
| **MPNST** | 11 | 6.7 | 6 | 3.0 |
| **MFS** | 28 | 17.0 | 52 | 25.6 |
| **MLPS** | 11 | 6.7 | 27 | 13.3 |
| **SS** | 10 | 6.1 | 6 | 3.0 |
| **UPS** | 43 | 26.1 | 70 | 30.4 |
| **WDLPS** | 5 | 3.0 | 5 | 3.0 |
| **other (<5)** | 21 | 12.7 | 23 | 11.3 |
| **unknown** | 1 | 0.6 | 0 | 0.0 |
| **Surgical margins reported differently for Manchester and VORTEX-Biobank cohorts*  *WHO PS = World Health Organisation performance status; DDLPS = de-differentiated liposarcoma; EMCS = extraskeletal myxoid chondrosarcoma; LMS = leiomyosarcoma; LPS = liposarcoma; MPNST = malignant peripheral nerve sheath tumour; MFS = myxofibrosarcoma; MLPS = myxoid liposarcoma; SS = synovial sarcoma; UPS = undifferentiated pleomorphic sarcoma; WDLPS = well-differentiated liposarcoma.* | | | | |

#### Supplementary table 3. RNA quality control for the main validation cohorts.

|  | **Manchester n=127** | **VORTEX-Biobank n=159** |
| --- | --- | --- |
| **Qubit yield (ng/µl)** | 154.7 | 127.9 |
| **RIN** | 2.2 | 2.2 |
| **DV200 (%)** | 45.3 | 47.6 |
| **Samples passed** | 126/127 (99%) | 154/159 (97%) |
| **Gene fails/sample** | 0.10 | 0.14 |
| **Block age (years)** | 12 | 7 |
| **Hypoxia-high result** | 53/126 (42%) | 70/154 (45%) |
| *RIN = RNA integrity number, DV200 = percentage of RNA fragments >200 bases* | | |

#### Supplementary table 4. Intra-tumour heterogeneity cohort samples.

| **Tumour** | **Sample** | **Histology** | **Yield (ng/µl)** | **RIN** | **DV200** | **% Tumour** |  |
| --- | --- | --- | --- | --- | --- | --- | --- |
| A | 1 | MLPS | 39.1 | 2.0 | 56 | 35-40 |  |
|  | 2 |  | 41.7 | 1.9 | 57 | 50-60 |  |
|  | 3 |  | 60.6 | - | - | 85-95 |  |
|  | 4 |  | 55.5 | - | - | 95 |  |
|  | 5 |  | 38.7 | 2.0 | 54 | 40 |  |
| B | 1 | MLPS | 33.8 | 2.6 | 17 | * |  |
|  | 2 |  | 23.0 | 2.5 | 18 | * |  |
|  | 3 |  | 63.8 | 2.5 | 25 | * |  |
| C | 1 | UPS | 114.0 | 2.4 | 36 | * |  |
|  | 2 |  | 118.0 | 2.4 | 36 | * |  |
|  | 3 |  | 47.4 | 2.3 | 28 | * |  |
|  | 4 |  | 58.8 | 2.3 | 37 | * |  |
| D | 1 | LMS | 288.0 | 1.0 | 52 | 75-80 |  |
|  | 2 |  | 201.0 | 1.0 | 61 | 70 |  |
|  | 3 |  | 101.4 | 1.9 | 60 | 80 |  |
|  | 4 |  | 154.2 | 2.3 | 60 | 75 |  |
|  | 5 |  | 343.0 | 1.4 | 59 | 75 |  |
|  | 6 |  | 445.0 | 1.0 | 63 | 80 |  |
|  | 7 |  | 127.5 | - | - | 80-85 |  |
|  | 8 |  | 198.0 | - | - | 80 |  |
| E | 1 | UPS | 37.9 | - | - | 50 |  |
|  | 2 |  | 31.3 | 2.1 | 50 | 35 |  |
|  | 3 |  | 35.1 | 2.1 | 47 | 25-30 |  |
|  | 4 |  | 63.3 | - | - | 35-40 |  |
| F | 1 | LMS | 60.3 | 2.1 | 23 | 75-85 |  |
|  | 2 |  | 115.5 | 2.3 | 25 | 60 |  |
|  | 3 |  | 84.3 | 2.9 | 43 | 90 |  |
|  | 4 |  | 182.1 | 2.7 | 22 | 85 |  |
|  | 5 |  | 52.3 | 1.8 | 44 | >90 |  |
|  | 6 |  | 31.9 | n/a | 47 | 90 |  |
| G | 1 | UPS | 127.8 | 2.3 | 58 | >90 |  |
|  | 2 |  | 110.1 | 2.3 | 52 | >90 |  |
|  | 3 |  | 150.6 | 2.4 | 52 | >90 |  |
|  | 4 |  | 110.4 | 2.1 | 54 | >90 |  |
| H | 1 | UPS | 20.0 | 2.4 | 19 | * |  |
|  | 2 |  | 21.4 | 2.5 | 15 | 85 |  |
|  | 3 |  | 20.5 | 2.4 | 18 | 80 |  |
|  | 4 |  | 45.4 | 5.2 | 22 | 85 |  |
|  | 5 |  | 28.8 | 4.3 | 33 | 90 |  |
| I | 1 | LPS | 33.3 | 2.7 | 33 | 85 |  |
|  | 2 |  | 116.4 | 3.0 | 37 | * |  |
|  | 3 |  | 71.7 | n/a | 32 | 35 |  |
| J | 1 | LMS | 222.6 | 2.4 | 14 | 90 |  |
|  | 2 |  | 195.9 | 2.4 | 11 | 85 |  |
|  | 3 |  | 186.9 | 2.5 | 11 | 75 |  |
| **no H+E available*  *MLPS = myxoid liposarcoma; UPS = undifferentiated pleomorphic sarcoma; LMS = leiomyosarcoma; LPS = liposarcoma* | | | | | | | |

#### Supplementary table 5. Differential expression of the 24-gene signature under hypoxia *in vitro* and *in vivo*.

| **Gene** | ***In vitro* 21% vs. 1% O_2_** | | | | **Protein marker neg vs. pos tumours** | | | | **Hypoxia-low vs. hypoxia-high tumours** | | | |
| --- | --- | --- | --- | --- | --- | --- | --- | --- | --- | --- | --- | --- |
|  | **HT1080** | | **SKUT1** | | **HIF-1α n=136** | | **CAIX n=152** | | **Manchester n=126** | | **VORTEX-Biobank n=154** | |
|  | **Mean Difference** | **Adjusted p** | **Mean Difference** | **Adjusted p** | **Mean Difference** | **Adjusted p** | **Mean Difference** | **Adjusted p** | **Mean Difference** | **Adjusted p** | **Mean Difference** | **Adjusted p** |
| *ALDOC* | **2**.**78** | **1**.**40E-05** | **3**.**00** | **1**.**00E-06** | **0**.**86** | **2**.**56E-03** | **0**.**98** | **1**.**80E-05** | 0.44 | 1.25E-01 | **0**.**81** | **1**.**65E-04** |
| *ANG* | **1**.**95** | **1**.**73E-04** | **1**.**04** | **2**.**71E-04** | -0.29 | 1.77E-01 | -0.09 | 2.37E-01 | **-0**.**80** | **2**.**78E-04** | -0.42 | 1.21E-02 |
| *ANKRD37* | **2**.**23** | **6**.**60E-05** | **3**.**25** | **9**.**00E-06** | **0**.**63** | **1**.**16E-03** | **0**.**55** | **1**.**51E-04** | **0**.**47** | **9**.**54E-03** | **0**.**75** | **1**.**00E-06** |
| *BHLHE40* | **1**.**30** | **2**.**20E-04** | **2**.**61** | **3**.**60E-05** | 0.39 | 9.39E-02 | **0**.**59** | **3**.**30E-03** | 0.20 | 2.94E-01 | **0**.**52** | **5**.**88E-03** |
| *BNIP3* | **3**.**17** | **1**.**40E-05** | **2**.**75** | **1**.**70E-05** | **1**.**05** | **4**.**25E-04** | **0**.**65** | **1**.**04E-03** | **0**.**93** | **1**.**40E-04** | **1**.**19** | **1**.**00E-06** |
| *BNIP3L* | **2**.**18** | **7**.**00E-06** | **2**.**76** | **7**.**00E-06** | 0.30 | 2.85E-02 | **0**.**37** | **2**.**90E-04** | -0.16 | 1.44E-01 | **0**.**39** | **6**.**70E-05** |
| *CDK18* | **2**.**69** | **4**.**31E-03** | **0**.**60** | **6**.**07E-03** | -0.03 | 5.84E-01 | -0.59 | 1.45E-02 | -0.57 | 4.53E-02 | **-0**.**79** | **1**.**52E-03** |
| *DDIT4* | **1**.**95** | **6**.**53E-04** | **1**.**79** | **7**.**10E-05** | 0.35 | 1.34E-01 | 0.32 | 5.26E-02 | 0.36 | 7.96E-02 | **0**.**73** | **1**.**72E-04** |
| *ENO2* | **2**.**20** | **2**.**10E-05** | **2**.**69** | **4**.**50E-05** | 0.63 | 5.41E-02 | **1**.**35** | **1**.**00E-06** | **1**.**18** | **1**.**00E-06** | **1**.**68** | **1**.**00E-06** |
| *FAM162A* | **2**.**66** | **1**.**30E-05** | **2**.**46** | **9**.**00E-06** | 0.35 | 2.42E-02 | **0**.**70** | **1**.**00E-06** | **0**.**39** | **8**.**58E-03** | **0**.**51** | **1**.**30E-05** |
| *GBE1* | **1**.**52** | **1**.**30E-05** | **1**.**57** | **4**.**70E-05** | 0.12 | 4.10E-01 | 0.14 | 1.52E-01 | 0.44 | 2.56E-02 | **0**.**50** | **6**.**68E-04** |
| *INSIG2* | **1**.**85** | **4**.**40E-05** | **1**.**31** | **1**.**97E-04** | 0.07 | 4.59E-01 | 0.26 | 1.21E-02 | -0.01 | 6.51E-01 | 0.17 | 5.10E-02 |
| *MXI1* | **1**.**90** | **3**.**90E-05** | **1**.**13** | **3**.**40E-04** | 0.06 | 4.95E-01 | 0.02 | 2.99E-01 | -0.25 | 7.74E-02 | 0.07 | 2.07E-01 |
| *NDRG1* | **3**.**24** | **7**.**00E-06** | **4**.**69** | **9**.**00E-06** | 0.58 | 5.41E-02 | **1**.**53** | **1**.**00E-06** | 0.12 | 4.42E-01 | **1**.**38** | **1**.**00E-06** |
| *P4HA1* | **2**.**04** | **4**.**70E-05** | **2**.**29** | **9**.**00E-06** | **0**.**55** | **1**.**34E-03** | **0**.**64** | **3**.**00E-06** | 0.29 | 4.53E-02 | **0**.**80** | **1**.**00E-06** |
| *PDK1* | **1**.**60** | **5**.**70E-05** | **2**.**85** | **1**.**70E-05** | **0**.**55** | **1**.**34E-03** | **0**.**61** | **5**.**00E-06** | **0**.**51** | **1**.**64E-03** | **0**.**72** | **1**.**00E-06** |
| *PFKFB4* | **1**.**57** | **2**.**10E-05** | **4**.**11** | **3**.**00E-05** | **0**.**79** | **1**.**34E-03** | **1**.**11** | **1**.**00E-06** | **0**.**91** | **1**.**00E-05** | **1**.**19** | **1**.**00E-06** |
| *PPFIA4* | **2**.**98** | **1**.**73E-04** | **2**.**29** | **2**.**36E-04** | 0.57 | 9.20E-02 | **0**.**94** | **4**.**36E-04** | 0.65 | 2.56E-02 | 0.00 | 3.34E-01 |
| *PRSS53* | **1**.**24** | **4**.**80E-05** | **0**.**77** | **1**.**39E-03** | 0.27 | 9.39E-02 | 0.32 | 1.13E-02 | -0.03 | 5.92E-01 | 0.02 | 3.06E-01 |
| *SLC2A1* | **1**.**12** | **1**.**90E-05** | **1**.**72** | **7**.**20E-05** | **1**.**13** | **9**.**13E-04** | **1**.**57** | **1**.**00E-06** | **1**.**37** | **1**.**00E-06** | **1**.**63** | **1**.**00E-06** |
| *SLC2A3* | **1**.**19** | **5**.**60E-05** | **1**.**55** | **1**.**20E-05** | **0**.**78** | **5**.**75E-03** | **0**.**93** | **5**.**30E-05** | **0**.**80** | **1**.**05E-03** | **1**.**34** | **1**.**00E-06** |
| *VEGFA* | **0**.**45** | **7**.**06E-03** | **1**.**22** | **5**.**70E-05** | **0**.**71** | **3**.**82E-03** | **0**.**93** | **1**.**00E-06** | **0**.**98** | **1**.**50E-05** | **1**.**05** | **1**.**00E-06** |
| *VLDLR* | **2**.**13** | **1**.**40E-05** | **2**.**07** | **1**.**75E-04** | -0.08 | 5.45E-01 | 0.11 | 2.52E-01 | -0.11 | 5.29E-01 | -0.41 | 6.23E-02 |
| *ZNF395* | **2**.**09** | **4**.**50E-05** | **2**.**17** | **7**.**00E-05** | 0.10 | 4.57E-01 | **0**.**51** | **7**.**52E-04** | -0.30 | 7.96E-02 | 0.25 | 4.49E-02 |
| **Genes upregulated** | **24** | | **24** | | **9** | | **16** | | **10** | | **17** | |
| *Benjamini (two stage) method used to correct for multiple t-tests, false discovery rate (Q) = 1%. Significant p-values for upregulated genes shown in bold.* | | | | | | | | | | | | |

#### Supplementary table 6. Univariate and multivariate survival analyses in the combined nanoString cohort.

|  | **Combined nanoString cohort (n=280)** | | | |  |
| --- | --- | --- | --- | --- | --- |
|  | **Univariate** | | **Multivariate** | |  |
|  | **HR (95% CI)** | **p** | **HR (95% CI)** | **p** |  |
| **Local recurrence free survival** |  |  |  |  |  |
| **Hypoxia** | **2**.**17 (1**.**01-4**.**68)** | **0**.**04** |  |  |  |
| **Age** | 1.01 (0.99-1.04) | 0.30 |  |  |  |
| **Sex** | 1.72 (0.75-3.93) | 0.20 |  |  |  |
| **WHO PS** | 0.97 (0.42-2.21) | 0.90 |  |  |  |
| **Size** | 1.01 (0.97-1.06) | 0.60 |  |  |  |
| **Grade** | 1.60 (0.67-3.78) | 0.30 |  |  |  |
| **Depth** | 3.32 (0.79-14.02) | 0.08 |  |  |  |
| **Histology** |  |  |  |  |  |
| **LMS** | - | - |  |  |  |
| **MFS** | 2.33 (0.30-18.2) | 0.42 |  |  |  |
| **MLPS** | 0.54 (0.03-8.65) | 0.66 |  |  |  |
| **MPNST** | 1.98 (0.18-21.8) | 0.58 |  |  |  |
| **SS** | 2.02 (0.26-15.8) | 0.50 |  |  |  |
| **UPS** | 2.38 (0.22-26.2) | 0.48 |  |  |  |
| **Other** | 0.25 (0.02-4.06) | 0.33 |  |  |  |
| **Metastasis free survival** |  |  |  |  |  |
| **Hypoxia** | **1**.**92 (1**.**28-2**.**87)** | **0**.**001** | **1.71 (1.11-2.63)** | **0.014** |  |
| **Age** | 1.00 (0.99-1.02) | 0.80 |  |  |  |
| **Sex** | 1.05 (0.70-1.59) | 0.80 |  |  |  |
| **WHO PS** | 1.03 (0.67-1.59) | 0.90 |  |  |  |
| **Size** | 1.02 (1.00-1.04) | 0.05 |  |  |  |
| **Grade** | **2.38 (1.42-3.98)** | **0.001** | **2.12 (1.25-3.60)** | **0.006** |  |
| **Depth** | **1**.**88 (1**.**03-3**.**45)** | **0**.**04** | **2.03 (1.08-3.82)** | **0.03** |  |
| **Histology** |  |  |  |  |  |
| **LMS** | **-** | **-** |  |  |  |
| **MFS** | 1.04 (0.39-2.72) | 0.94 |  |  |  |
| **MLPS** | 0.52 (0.15-1.80) | 0.30 |  |  |  |
| **MPNST** | 0.35 (0.07-1.81) | 0.21 |  |  |  |
| **SS** | 1.49 (0.58-3.80) | 0.40 |  |  |  |
| **UPS** | 0.95 (0.25-3.53) | 0.94 |  |  |  |
| **Other** | 0.99 (0.37-2.65) | 0.99 |  |  |  |
| **Disease free survival** |  |  |  |  |  |
| **Hypoxia** | **2**.**00 (1**.**35-2**.**95)** | **0**.**0004** | **1.86 (1.24-2.81)** | **0.003** |  |
| **Age** | 1.00 (0.99-1.02) | 0.50 |  |  |  |
| **Sex** | 1.12 (0.75-1.67) | 0.60 |  |  |  |
| **WHO PS** | 1.16 (0.77-1.75) | 0.50 |  |  |  |
| **Size** | 1.02 (1.00-1.04) | 0.06 |  |  |  |
| **Grade** | 2.06 **(1**.**28-3**.32**)** | **0**.**003** | **1.76 (1.08-2.86)** | **0.02** |  |
| **Depth** | 1.77 (1.00-3.10) | 0.05 |  |  |  |
| **Histology** |  |  |  |  |  |
| **LMS** | - | - |  |  |  |
| **MFS** | 0.99 (0.41-2.40) | 0.97 |  |  |  |
| **MLPS** | 0.43 (0.13-1.41) | 0.16 |  |  |  |
| **MPNST** | 0.62 (0.17-2.20) | 0.46 |  |  |  |
| **SS** | 1.32 (0.60-3.13) | 0.52 |  |  |  |
| **UPS** | 0.78 (0.22-2.77) | 0.70 |  |  |  |
| **Other** | 0.83 (0.33-2.06) | 0.68 |  |  |  |
| **Overall survival** |  |  |  |  |  |
| **Hypoxia** | **2**.**38 (1**.**57-3**.**62)** | **0**.**00003** | **2.24 (1.42-3.53)** | **0.00096** |  |
| **Age** | **1**.**02 (1**.**00-1**.**03)** | **0**.**01** | 1.01 (1.00-1.03) | 0.35 |  |
| **Sex** | 1.36 (0.89-2.09) | 0.20 |  |  |  |
| **WHO PS** | 1.35 (0.88-2.08) | 0.20 |  |  |  |
| **Size** | **1.02 (1.00-1.04)** | **0.02** | 1.01 (0.99-1.03) | 0.25 |  |
| **Grade** | **2.13 (1.28-3.54)** | **0.003** | 1.67 (0.98-2.85) | 0.18 |  |
| **Depth** | 1.48 (0.84-2.62) | 0.20 |  |  |  |
| **Histology** |  |  |  |  |  |
| **LMS** | **-** | **-** | - | - |  |
| **MFS** | 0.62 (0.28-1.40) | 0.25 | 0.61 (0.26-1.45) | 0.26 |  |
| **MLPS** | **0.20 (0.05-0.75)** | **0.02** | 0.36 (0.09-1.46) | 0.15 |  |
| **MPNST** | 0.45 (0.14-1.51) | 0.20 | 0.60 (0.17-2.15) | 0.43 |  |
| **SS** | 0.90 (0.42-1.94) | 0.79 | 0.71 (0.31-1.65) | 0.43 |  |
| **UPS** | 0.29 (0.06-1.35) | 0.11 | 0.41 (0.08-2.05) | 0.28 |  |
| **Other** | 0.67 (0.29-1.52) | 0.34 | 0.67 (0.28-1.62) | 0.37 |  |
| *P-values are from the log-rank test.*  *WHO PS = World Health Organization performance status; LMS = leiomyosarcoma; MFS = myxofibrosarcoma; MLPS = myxoid liposarcoma; MPNST = malignant peripheral nerve sheath tumour; SS = synovial sarcoma; UPS = undifferentiated pleomorphic sarcoma; other = other histology with <5 cases in total.*  *Age and size were analysed as continuous variables. Hypoxia, sex, WHO PS, grade, depth and histology were analysed as categorical variables. Hypoxia – high versus low; Sex – male versus female; WHO PS - 1/2 versus 0; Grade – I/II versus III; Depth – deep versus superficial. Histology – each versus LMS.* | | | | | |
